# Covid-19 related excess mortality: An analysis by age for selected countries

**DOI:** 10.1371/journal.pone.0353766

**Published:** 2026-07-16

**Authors:** David Madden

**Affiliations:** School of Economics, University College Dublin, Dublin, Ireland; University of Karachi, PAKISTAN

## Abstract

**Background:**

Measures of excess mortality are generally regarded as providing the best metric for countries’ experience during the Covid pandemic of 2020–2022. Such measures, however, need to be adjusted to take account of the different age structures of different countries as Covid-related mortality can differ substantially by age.

**Methods:**

This paper analyses excess mortality data for a selection of countries using the Short Term Mortality Fluctuations dataset. A standardised age distribution is applied to the data to obtain age-adjusted excess mortality and this is compared to crude excess mortality data. The metric of years of life lost (YLL) is also calculated for the same dataset and cluster analysis is applied to investigate relationships between the mortality metrics and GDP per capita and inequality.

**Results:**

The age adjustment makes a significant difference for some countries but the overall ranking of countries in terms of excess mortality changes very little, with rank correlation coefficients in excess of 0.95 for the metrics of crude mortality, age adjusted mortality and YLL Age adjusted excess mortality has a rank correlation of −0.69 with GDP per capita but the rank correlation with the Gini coefficient of 0.11 is not statistically significant.

**Conclusions:**

Rankings of countries by excess mortality during the Covid period are robust to age adjustment and choice of mortality metric. Cluster analysis suggests distinct clusterings of countries with similar outcomes, particularly the former planned economies of central and eastern Europe.

## Introduction

The Covid-19 pandemic started in China in November 2019, with the first large outbreak observed in Wuhan in December. Over the following months it spread to most of the world and in March 2020 the World Health Organisation (WHO) declared it a pandemic, i.e., the widespread transmission of an infectious disease over a large region of the world. Most countries responded to the arrival of Covid with various public health measures, including orders to “stay at home”, curtailment of public transport and other measures to reduce the spread of disease, measures which came to be known by the general phrase “lockdown”.

The severity of lockdown varied by country (e.g., Sweden notably introduced fewer restrictions) and also by time, in that when case numbers started to fall in early summer 2020 many of the restrictions were lifted. There were subsequent waves of Covid, mostly associated with the arrival of new variants, in nearly all cases more transmissible than earlier variants and in some cases with higher fatality rates, and in many countries restrictive measures were re-introduced. At the same time however, vaccines were developed with remarkable speed. These vaccines either prevented infection (though not transmission) or at least ensured that conditional on being infected, survival rates were considerably higher. There were also breakthroughs in treatments like Paxlovid. By around mid-2022 lockdown measures had been greatly eased in many countries and there was a sense by end-2022 that the worst of the pandemic was “over”.

Arguably, it is incorrect to regard the pandemic as over, given that there are still cases of infections and mortality but one “official” indication that some sort of turning point had been reached was the statement by the WHO in May 2023 that Covid had changed from being a public health emergency to now being an ongoing public health issue. For a comprehensive source on Covid related issues, see https://www.who.int/health-topics/coronavirus#tab=tab_1.

In this paper, we look at measures of excess mortality over the “Covid period”, defined as from the start of 2020 to the end of 2022. Intuitively, excess mortality is the difference between actual mortality and a measure of projected mortality, where projected mortality is the counterfactual of what mortality would have been in the absence of unexpected events, such as Covid. As explained in more detail below, this measure has the advantage of not just taking account of higher mortality arising directly from Covid, but also of taking account of changes in mortality which *indirectly* arise from Covid, e.g., due to delayed diagnosis and treatment of other causes, or fewer traffic related accidents etc.

This paper makes two principal additions to this literature. First, it builds upon a set of studies which examine Covid excess mortality using a variety of different metrics and this literature is reviewed below. Second, it applies hierarchical cluster analysis to identify groups of countries in terms of their excess mortality and two other relevant population-level measures of economic achievement, GDP per capita and the Gini coefficient. To the best of our knowledge, ours is the only paper taking such an approach. This provides a very useful typology of countries in terms of a key Covid-related outcome and relevant economic outcomes and could be used in terms of identifying the particular risks facing countries should there be future pandemics. In the remainder of this introduction we briefly review other relevant papers in this area.

Dating from around 2022, a number of papers have analysed cross-country patterns for various Covid-related metrics. One of the first of these was from the World Health Organisation (WHO) [1] who discussed some of the data and methodological challenges involved in calculating excess mortality and provided monthly figures for 2020–2021. Similar to our paper here, they were restricted in their choice of sample by the lack of reliable data, leading them to have to estimate not just excess mortality but also all cause mortality for some countries.

Levitt et al [2] also compared measures of excess mortality from a number of different sources, such as the WHO and *The Economist.* Similar to the WHO study, they limit their analysis to data for 2020 and 2021 and their sample of countries is also limited by data availability. Nevertheless, they find high correlation across most of the metrics analysed with Pearson correlation coefficients in excess of 0.97.

Rousson and Locatelli [3] focus on years of life lost as their Covid measure and present data for a sample of 30 countries but just for the year 2020. Their study focuses on a comparison of Covid metrics but all taking the general “years of life lost” type approach, and similar to other studies mentioned they find correlation coefficients (Spearman rank correlations in this case) in excess of 0.95.

One feature which can critically affect mortality metrics is making the appropriate adjustment for age (and it is a central feature of this paper). Such age adjustments are particularly important when analysing the effect of a condition such as Covid, where mortality is not uniform across the age distribution. Ullrich-Kniffka and Schöley [4] and Pallari et al [5] are two examples of papers with a particular emphasis on this issue. The former paper focuses on making age adjustments to P-scores while the latter looks at total, sex- specific and age- specific excess mortality for multiple countries for the whole years 2020 and 2021. Pallari et al also applied multilevel regressions to investigate the association between Covid related mortality and other country factors such as the Human Development Index (HDI), the Gini coefficient and other health morbidities, an approach which is in the spirit of the cluster analysis applied in this paper, even if using a slightly different methodology.

The direct application of cluster analysis to Covid mortality is less common. Tafti et al [6] applied k-means clustering to the trend of daily new infections for a sample of 16 countries across Europe, the Americas and Asia and found two clusters, one of high incidence and one of low incidence. However they did not include any other country level variables such as GDP per head or the Gini coefficient. Hasankhani et al [7] applied Latent Class Growth Modelling and Growth Mixturte Modelling to mortality estimates for a wider sample of countries for the 2020–2022 period, but again did not include other population-level variables.

Overall, our review of these papers illustrate that comparative analysis of Covid mortality metrics has tended to focus on (a) different estimates of excess mortality (b) different metrics such as years of life lost and (c) the impact of age adjustment. However, to the best of our knowledge there is no paper which addresses all of these issues simultaneously. The contribution of the present paper is to bring these strands together and to also consider other population level economic outcomes via cluster analysis. Like many of the papers cited, the range of countries analysed is limited by data availability. In terms of the remainder of the paper, in section 2 we discuss how to measure excess mortality in more detail and review and compare the principal estimates available. Our principal aim is to examine excess mortality on an age-adjusted basis and how it varies by age group and by country. This means having to choose a specific estimate of excess mortality and this is covered in section 2.

In section 3 we review excess mortality for all ages and then look more closely at mortality rates by specific ages. As fatality rates differ substantially by age, part of the difference between countries overall mortality rates may be explained by the different age structure of the population. We therefore examine what excess mortality rates for our selection of countries would have been if they had all had a “standard” age structure and compare the rankings of countries by crude excess mortality and age adjusted excess mortality.

In section 4 we examine a different measure of mortality, years of life lost and we also analyse age specific mortality rates. Section 5 investigates how age adjusted excess mortality rates varied with population-level measures such as GDP per head and within country inequality and we also apply hierarchical cluster analysis to see if distinct clusters of countries can be identified. Section 6 presents concluding remarks.

## Methods

### Study design

This study is secondary analysis of quantitative data collected by Karlinsky and Kobak [8] and provided in the Human Mortality Database (see http://mortality.org), a joint venture between the University of California, Berkeley and Max Planck Institute for Demographic Research. This data is available in *Our World in Data*.

### Ethics

As this study comprises secondary analysis of aggregate country data no ethical approval was required.

### Measurement

The principal measurement analysed in this study is excess mortality. The use of excess mortality statistics as a means of monitoring mortality from Covid 19 arose from a general dissatisfaction with the use of conventional statistics on cause of death, and this applies not just to Covid 19 but also other infections such as influenza.. This can occur owing to problems with diagnosis and cause-of-death coding as well as interactions with pre-existing conditions, such as cardiovascular problems [9]. Such interactions can complicate the classification of cause of death, as it could reasonably be attributed to a pre-existing condition or Covid.

Owing to these limitations, all-cause excess mortality is now widely used as a metric of mortality from the pandemic. It compares weekly or monthly specific death rates over a period of time with some projected mortality level. Quite how to calculate this projected mortality level is a modelling choice and discussed in detail below. Given projected mortality however, excess deaths can then be expressed as


$$\begin{array}{c} Excess\;deaths=Reported\;deaths-Projected\;deaths \end{array}$$

They can also be expressed via P-scores, which are more comparable across countries as they take account of different underlying population sizes:


$$\begin{array}{c} P-score=\frac{Reported\;deaths-Projected\;deaths}{Projected\;deaths} \end{array}$$

Karlinsky and Kobak [8], henceforth KK, provide a very useful conceptual breakdown of excess mortality for the Covid 19 period:

Excess Mortality = (A) Deaths caused directly by Covid
  + (B) Deaths caused by medical system failures due to Covid
  + (C) Excess Deaths from Other Natural Causes
  + (D) Excess Deaths from Unnatural Causes
  + (E) Excess Deaths from Extreme Causes (wars, heatwaves etc).

Factor (B) may arise if services for other conditions are under-resourced, thereby resulting in higher mortality. The contribution of factor (C) may in some cases be negative. For example, if preventative Covid measures also reduce the transmission of other infectious diseases such as influenza, then excess deaths from these diseases could be negative [e.g., 10]. Factor (D) includes mortality arising from traffic accidents, homicides, suicides and drug overdoses and accidents [see 11], for an analysis of these issues for the US for the March-August 2020 period). Factor (E) is not an issue for many countries. For those analysed here, the effect of the August 2020 heatwave in Europe is explicitly accounted for in the numbers used.

KK speculate that (C) is likely to be negative, while D and B are both small (for developed countries at least), suggesting that excess mortality is likely to provide a reasonable lower-bound to “true” Covid deaths.

Nepomuceno et al [12] discuss the sensitivity of excess deaths to a number of different assumptions/choices made: these include the choice of mortality index (absolute or relative), how to calculate projected deaths, the number of years included in the reference period from which projected deaths are calculated and the time unit of the death data. We now discuss each of these in turn.

#### Choice of mortality index.

The choice of mortality index boils down to whether mortality is expressed in terms of actual numbers, or in terms of rates with respect to some underlying base, e.g., in the formulation above they are expressed with respect to projected deaths, but they could also be expressed with respect to population. The use of projected deaths as the base takes account of the age structure of the population, unlike the use of the crude population. For example, Msemburi et al [1] point out that in 2019 both Germany and Iran had similar populations, around 83 million. Mortality in Germany was around 2.5 times higher however, as Germany has an older population with many more people aged over 65. We accordingly present our results below initially with respect to crude underlying population, but then relative to a standardised age distribution, thus controlling for age structure. We also present age-specific excess mortality rates for different age groups.

#### Choice of model.

The model chosen to calculate projected deaths is arguably the most critical decision to be made. One of the simplest models is a period specific-average model, whereby projected deaths (or more accurately baseline deaths in this case) are simply the average for the previous *n* years (values of 3, 5 or 10 would be common). This model may be employed on an overall or age-specific basis and can be augmented with other features. For example, time trends (either overall or age-specific) and/or harmonic terms which take account of seasonality can be added.

More sophisticated models are also possible, including those that explicitly account for the nature of mortality data. Since death is a binary variable (dead or not) it follows a Bernoulli distribution, which in turn can be approximated by a Poisson or Negative Binomial distribution (in conditions where the underlying number of “trials” is large relative to the probability of the event) which in turn can also take account of within year seasonal trends [for example, see the excess mortality estimates produced by the WHO, [1]].

Alternative prominent methodologies include the machine-learning approach adopted by *The Economist* (https://www.economist.com/graphic-detail/2021/05/13/how-we-estimated-the-true-death-toll-of-the-pandemic) and the model of The Institute for Health Metrics and Evaluation (IHME) published in *The Lancet* [13]. Later in this paper, we explain our choice of model and examine how our measures of excess mortality differ from some of the others.

#### Choice of reference period.

The reference period should ideally reflect the circumstances which would have applied in the absence of the pandemic. A short time period, immediately before the pandemic, has the advantage of being up-to-date and can capture recent mortality trends. However, if the time period is too short then it may contain a lot of “noise” which would then be incorporated into projected excess deaths. The typical choice of reference period for calculating excess mortality during the Covid 19 pandemic has been 2015–2019, though in some cases a longer, ten-year period has been chosen, when monthly data are not available.

#### Time unit.

The choice of time unit is typically between weekly or monthly, though in some cases only annual data may be available. Weekly data have the advantage of capturing more immediately any sudden change in mortality conditions. Generally, the longer the time unit, the more the data will be smoothed. It may be preferable to have high frequency data which the analyst can choose to smooth if they wish, rather than lower frequency data where the analyst may not know the degree to which it has been artificially smoothed. The data employed in this paper is weekly.

### Years of life lost (YLL)

Excess mortality data can be calculated on a crude or age-adjusted basis. However, when aggregating this data we effectively regard all deaths as “equal”. Whether someone dies aged 20 or 80 it is still regarded as one death. Another potential metric for mortality is “years of life lost”, which we abbreviate to YLL. On average someone aged 20 will presumably have many more potential years of life left compared to someone aged 80. Precisely how many can be obtained from life-tables. The IHME in its Global Burden of Disease (GBD) Study prepared a reference life-table for calculating YLL due to premature mortality [GBD, 2019] [14]. For example, they calculate that a premature death aged 20 implies a loss of life of approximately 69 years, while a premature death at 80 implies a loss of approximately 13 years. Therefore, in calculating an overall metric of mortality, the death of a 20 year old would have a higher weight (69/13 = 5.3) compared to the death of an 80 year old. It is important to note that this is not an “ethical” weight, suggesting that one age group is in some sense more “worthy” than another. It simply reflects a different metric for mortality.

### Statistical analysis

#### Excess mortality analysis.

The measures of excess mortality chosen here are based on Karlinsky and Kobak [8]. In their original study, they used the *World Mortality Dataset* for their measures of all-cause mortality. In this study however, we use the figures provided in the Human Mortality Database (see HMD and http://mortality.org), a joint venture between the University of California, Berkeley, and Max Planck Institute for Demographic Research [15]. They compiled the *Short Term Mortality Fluctuations (STMF)* dataset, which provides weekly data, broken down into five age groups, with starting dates ranging from as early as 1990 in some cases and all the way up to the present [16,17]. Such data are available for 36 countries (we restrict our analysis to a subset of these, since for Iceland, Luxembourg and Estonia some of the cell-sizes for age specific mortality were too small for analysis).

STMF provides data on actual mortality by age for this set of countries. To calculate excess mortality we also need projected deaths, either in absolute terms or via P-scores, and also broken down by age. The P-scores were obtained from Our World in Data based on the methodology outlined in Karlinsky and Kobak (2021, https://ourworldindata.org/excess-mortality-covid#excess-mortality-p-scores-by-age-group). To obtain baseline deaths, they fit the following regression model for each country and age-group:


$$\begin{array}{c} D_{t,Y}=\alpha_{t}+\beta Y+\epsilon \end{array}$$

where $D_{t,Y}$ is deaths observed in week *t* of year *Y*, β is the linear slope or trend across years and ϵ is an error term which is normally dis*t*ributed with mean zero and variance, $\sigma^{\text{2}}$. This model can capture time trends via β and seasonal fixed effects via $\alpha_{t}$. This model was fitted over the 2015–2019 period and in all cases the β term was statistically significant, showing the presence of a time trend and indicating that a specific period-average approach would not have been appropriate. The countries analysed here all had weekly data and so the model was estimated over 52 weeks. For 2020, when there was a 53^rd^ “week” the value for week 52 was used.

Then the baseline or projected deaths for any given year, say 2020, is $\hat{B_{t=\text{2}\text{0}\text{2}\text{0}}}=\hat{\alpha_{t}}+\hat{\beta }.\text{2}\text{0}\text{2}\text{0}$ and excess deaths for our period under review is then given by


$$\begin{array}{c} \Delta =\sum_{t\geq t_{\text{1}}}\left(D_{t,\;\text{2}\text{0}\text{2}\text{0}}-\hat{B_{t,\text{2}\text{0}\text{2}\text{0}}}\right)+\sum_{t\geq t_{\text{1}}}\left(D_{t,\;\text{2}\text{0}\text{2}\text{1}}-\hat{B_{t,\text{2}\text{0}\text{2}\text{1}}}\right)+\sum_{t\geq t_{\text{1}}}\left(D_{t,\;\text{2}\text{0}\text{2}\text{2}}-\hat{B_{t,\text{2}\text{0}\text{2}\text{2}}}\right) \end{array}$$

and we set $t_{\text{1}}=$ week 1 of January in 2020, 2021 and 2022.

On the basis of this approach, starting from week 1 of January 2020, P-scores by age group are available in Our World in Data for a selection of countries. In addition scores were also kindly provided to us by Dmitry Kobak for a small subset of countries which were not included in the Our World in Data website. These were Australia, Canada, South Korea, Sweden and Taiwan (only for 2020 and 2021). Therefore in total we have 32 countries for analysis, although for Taiwan we only have data for 2020 and 2021. Note that our sample of countries is not in any sense “representative”. We are restricted to those countries for whom P-scores are available for the years in question. Other studies such as Levitt et al [2], Rousson and Locatelli [3] also use relatively restricted samples of countries owing to data availability.

Our choice of the KK data is motivated by its having excess mortality estimates by age group and by week and for a reasonably wide (though not representative) sample of countries. However, before examining our excess mortality data, we first compare the figures based upon the KK approach with those provided by three other agencies: the WHO, *The Economist* and the IHME, sometimes referred to as *The Lancet* study [Msemberi et al, 2023 [1], The Economist, 2021 and [13]]. We do this as it is important to have confidence that our chosen measure is in some sense “reasonable” given that there are different methodologies in calculating excess mortality.

As discussed already, the WHO estimates are based upon a Negative Binomial model, allowing for within year seasonal variation, while *The Economist* uses a machine learning approach. The Lancet/IHME study uses an ensemble approach with six different models each fitted separately by location. The first four models were based upon estimating the seasonal pattern of mortality and then estimating the time trend not accounted for by seasonality. Two other models, one based upon a Poisson model with fixed effects for week and year and the other simply using the weekly mortality for 2019, were also estimated and the final estimated of projected deaths was a weighted ensemble with weights derived from how each model performed in an out of sample predictive validity test (the models were estimated on data up to March 2019 and then tested on the March 2019-February 2020 period).

**Fig 1:**
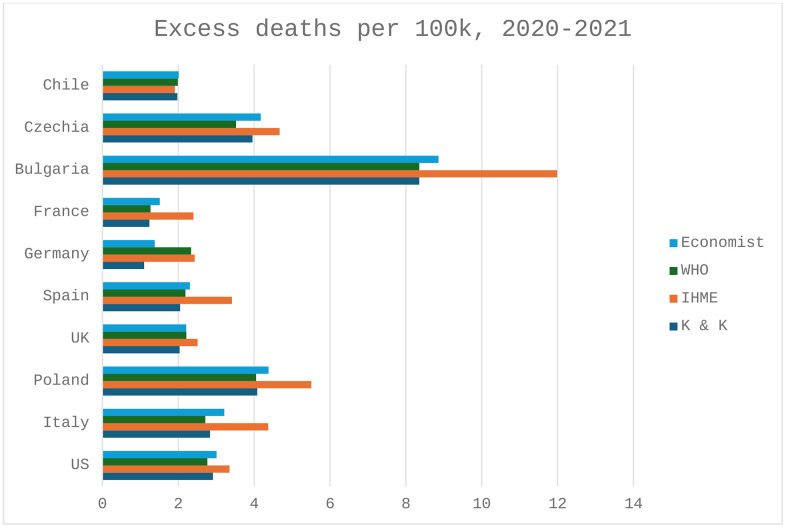
Excess Mortality 2020-2021 (per 100k) as Estimated by Different Models.

Thus before commencing our statistical analysis, it is important to verify that the KK excess morality estimates are comparable to other available estimated. Rather than compare results on a country by country basis, we identified the ten countries from our sample with the highest absolute number of cumulative excess deaths (note that the WHO and IHME results only extend as far as 2021, so we are examining the countries with highest excess mortality by end-2021). These were, in descending order: the United States, Italy, Poland, United Kingdom, Spain, Germany, France, Bulgaria, Czechia and Chile. Fig 1 presents bar charts of cumulative excess mortality, as a fraction of population, so as to enable comparability. Note that this does not take account of age structure. However, that is not important in this case as we are examining the within country variation in excess mortality estimates, based upon our four different models.

Visual inspection confirms that estimates from the IHME are considerably higher than for the other three models. For nine of the ten countries (the only exception is Chile), the IHME estimates are the largest, in some cases, e.g., Bulgaria, substantially so. The next highest estimates are from *The Economist*, and then third and fourth highest is shared between WHO and the estimates which we analyse in this paper (KK), though in fairness the differences between all the non-IHME estimates are very small.

The IHME estimates have been the subject of some criticism on the basis that they provide an overestimate of excess mortality. For example Bager et al [18] criticise the estimates for Denmark (although not included in Fig 1, the IHME estimates for 2020–2021 are up to ten times higher than those of the other three models reviewed here). Similarly, Moeti et al [19] criticise the estimates for Sub-Saharan Africa, again on the basis that they are too high [20] reply to these comments on behalf of IHME.) Given these criticisms it seems best to regard the IHME estimates as an outlier.

**Fig 2:**
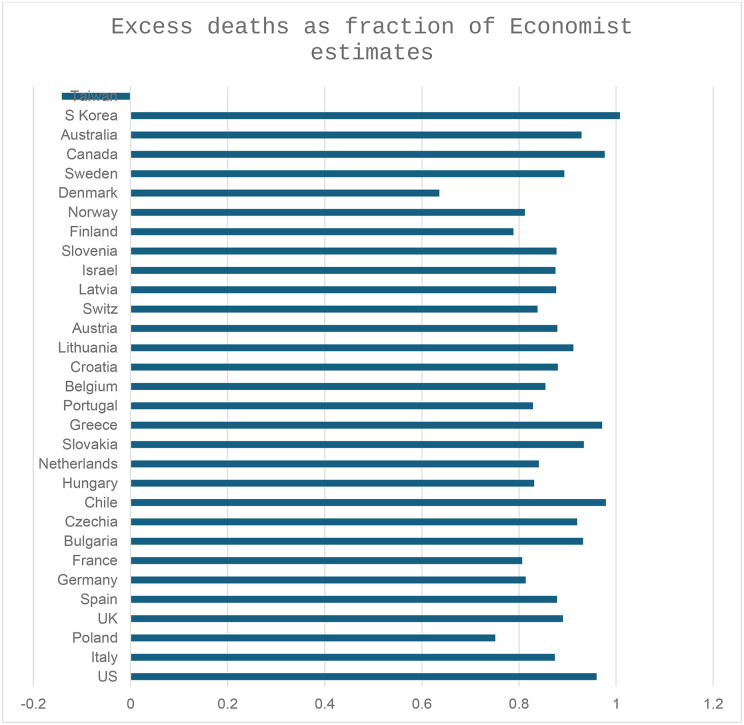
K&K Excess Mortality Estimates as Fraction of *The Economist*, 2020-2022.

Fig 2 compares estimates for the longer period, 2020–2022, between KK and *The Economist*, the only two models covering this period. Space permits us to include all of our countries in this case, and we present the KK estimates as a fraction of those of *The Economist*. Apart from South Korea, where the KK estimates are marginally higher than *The Economist*, the KK estimates are typically within 80 per cent of those of *The Economist*, the exceptions being Denmark, Finland and Poland.

Overall then, it seems fair to say that the estimates of excess mortality used here for subsequent analysis, those of KK, are of a similar order of magnitude to those produced by WHO and *The Economist*, if perhaps at the lower end. In addition, much of our analysis will concentrate on country *rankings*, and such rankings are very similar between the different measures. In no case does the rank correlation coefficient between any two of the measures fall below 0.98.

### Cluster analysis

In addition to calculating age-standardised excess mortality and YLL measures for our sample of countries, we also investigate the association between age standardised excess mortality and the level of GDP per capita and inequality as measured by the Gini coefficient of disposable income per head (results for other excess mortality metrics are available on request).. We do this by applying cluster analysis to investigate if a typology of country experiences can be identified.

Hierarchical cluster analysis proceeds via a series of successive fusions of the observations into groups (recall each observation is an excess mortality-GDP per capita pair or an excess-mortality-Gini pair, by country). Suppose initially there are *n* distinct pairs in the data. The first stage of cluster analysis fuses the two most similar pairs to form *n-1* clusters and this process continues. At each stage of the cluster analysis pairs which are most “similar” are fused into a group, with different approaches to fusing depending upon the different ways of defining similarity/distance between groups. In this paper the average-link method is used where the closest two groups are determined by the average (dis)similarity between the observations of the two groups at each step.

Ultimately, the fusion into clusters could proceed until the entire sample has been fused into one group. Hence some form of “stopping rule” is needed. There is little in the way of definitive advice on choice of stopping rule, and a mixture of statistical stopping rules and researcher discretion is usually employed [21]. The stopping rules employed here are the Calinski-Harabasz (CH) pseudo F, the Duda-Hart (DH) index and the pseudo T squared index.

Suppose there are *n* observations in total and *k* clusters. Then the CH index is given by $CH=\frac{\left(TSSD-\sum_{i=\text{1}}^{k}{SSD}_{i})/(k-\text{1}\right)}{\left(\sum_{i=\text{1}}^{k}{SSD}_{i})/(n-k\right)}$ where*TSSD* is the total sum of squared distances, and *SSD*_*i*_ is the sum of squared distances within group i. Effectively this compares the sum of squared distances between the clusters relative to the sum of squared distances within the clusters, adjusting for the number of clusters. If *CH* increases monotonically with *k* this is indicative of no natural clustering, in a sense there are “too many” clusters. If *CH* declines monotonically with *k* then again there is no clustering but this time for the opposite reason: there is only one cluster. However, if *CH* increases to a maximum at *k* and then decrease, this suggests the presence of k clusters.

The other stopping rules utilized are the Duda-Hart and pseudo T squared indices. Consider the case where we have k + 1 and k clusters and let $DH=\frac{{SSD}_{k+\text{1}}}{{SSD}_{k}}$ represent the sum of squared distances for the data with k + 1 clusters relative to the sum of squared distances with k clusters. As with the CH index, if a maximum at *k* is observed, then this suggests *k* clusters. Closely related to the DH index is the pseudo T squared index, which is the ratio of the between cluster sum of squares for *k* and *k + 1* to the sum of the within cluster sum of squares of *k* and *k + 1* clusters, adjusted for the number of observations in each cluster. In this case a lower value of the pseudo T squared index indicates the presence of clustering.

In common with other studies in this area subjective judgements on behalf of the researcher are also employed as the stopping rules can sometimes indicate an implausible number of clusters or a number which is not helpful in terms of subsequent analysis [21].

## Results

### Excess mortality

We first present results on excess mortality as a fraction of the underlying population using the KK approach. The underlying population figures are taken from the *United Nations World Population Prospects 2024* [22] As we are looking at cumulative excess mortality over the 2020–2022 period, we use average population over that period as our base.

**Fig 3:**
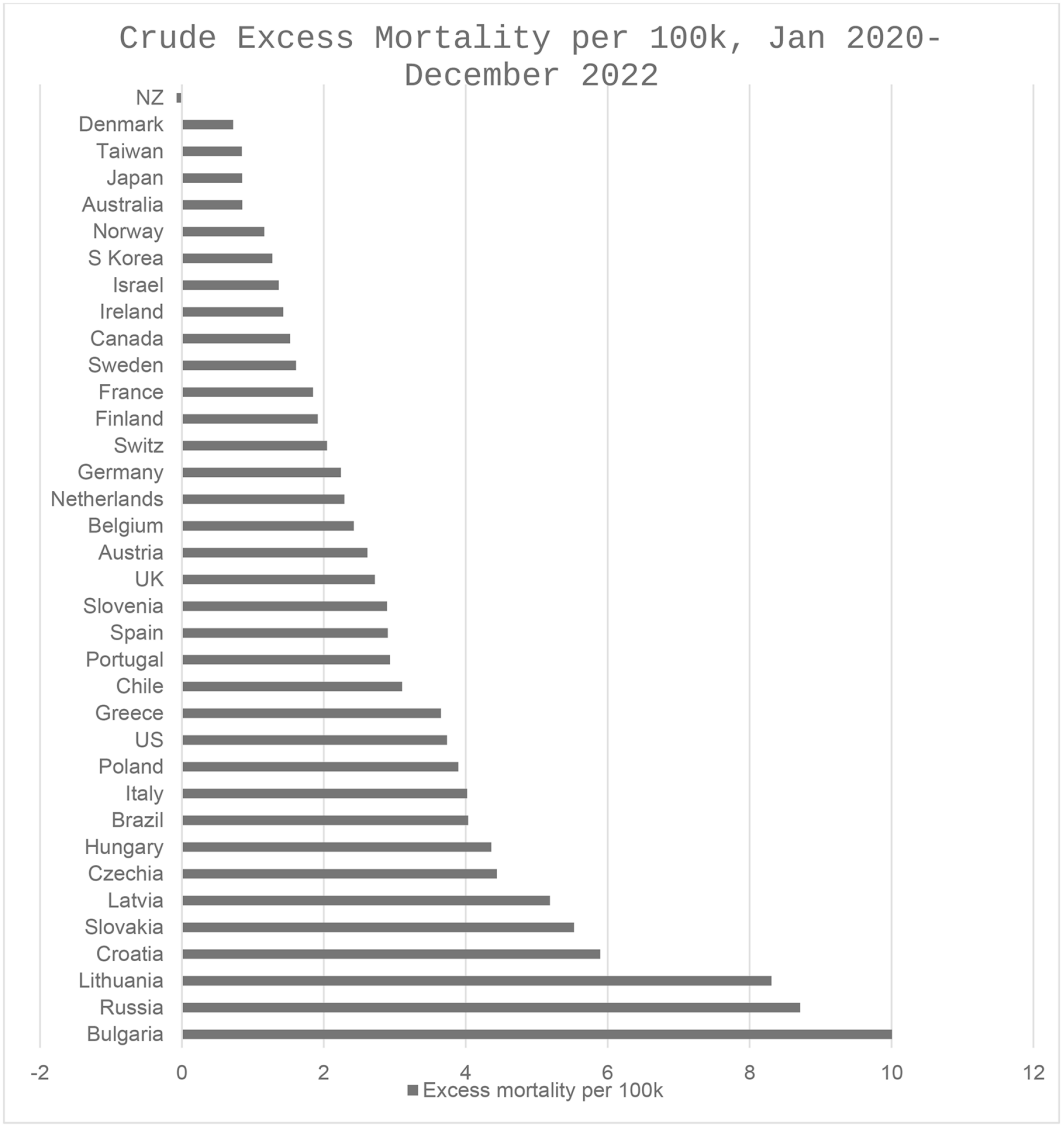
**Cumulative Excess Mortality per 100K of Population, 2020-** **2022.**

Fig 3 presents these cumulative excess mortality estimates for our selected countries.Note we also include some extra countries (Brazil, Ireland, Japan, Russia and Taiwan) where we have overall excess mortality estimates but not by age-group, and these countries will not feature in the age-adjusted analysis). New Zealand is estimated to have the lowest excess mortality with a *deficit* of actual deaths relative to projected deaths. The highest estimates are for Bulgaria, with excess mortality estimated at 10 per 100 thousand of the population.

Crude figures such as these can be misleading, however, as they do not take account of the fact that countries will differ in age-structure. We therefore calculate excess mortality controlling for age structure, by applying the age-specific mortality rates for each country to a common age distribution. The common age distribution we use is that provided by Eurostat [23], who provide a standardised age distribution based upon the EU-27 plus EFTA countries. For the age groups we consider (0–14, 15–64, 65–74, 75–84, > 84), the proportions are 0.16, 0.645, 0.105, 0.065 and 0.025 respectively.

**Fig 4:**
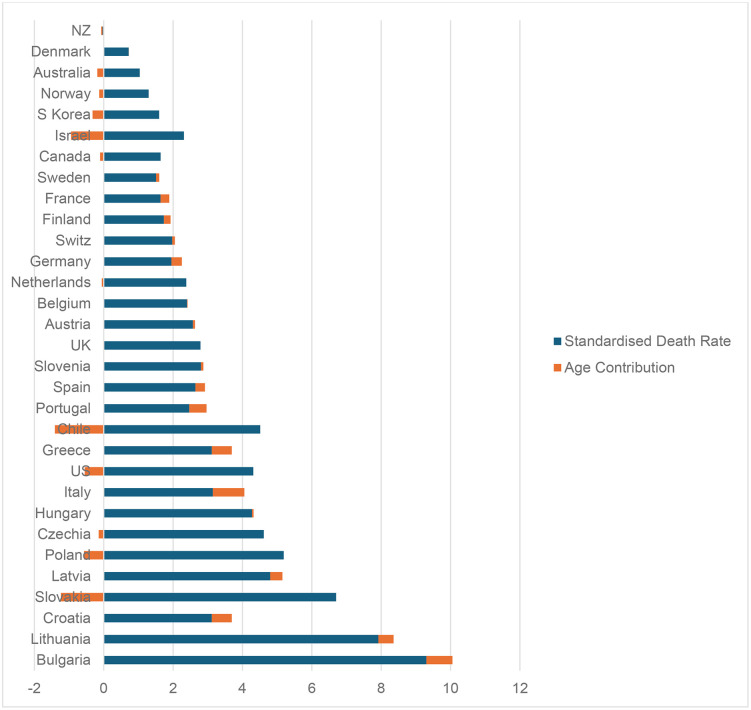
Age-standardised excess mortality rates per 100k.

In Fig 4, we show the age-standardised excess mortality rates for our selected countries and we also show what we label the “age-contribution”, that part of the crude excess mortality which is contributed by the specific age-structure of the country in question. For most of our countries the age-contribution is positive, i.e., it added to crude excess mortality and if the country had the standard Eurostat age structure then its crude excess mortality would have been lower.

**Fig 5:**
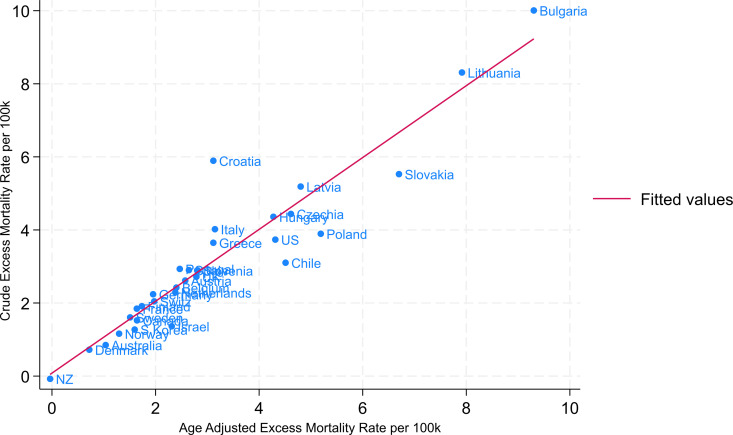
Age-adjusted excess mortality rate versus crude mortality rate.

Overall, standardisation has little effect on the rankings of countries in terms of excess mortality. The rank correlation coefficient between crude and standardised excess mortality is around 0.96 and is highly significant. Fig 5 shows essentially the same information via a scatter plot between crude excess mortality and age adjusted excess mortality.

### Age-specific mortality rates

Next we turn to examine the age-specific mortality rates to see which age groups fared best in which countries (table 1 in supplementary files S1 Table in S1 File). For a majority of our selected countries cumulative excess mortality in the 0–14 age group was *negative* over the period. This almost certainly reflects averted births. Most mortality in this age group happens at or very shortly after birth [24]. It is plausible that the Covid pandemic led people to defer planned pregnancies, perhaps owing to concerns over the quality of medical support available during a pandemic, or perhaps owing to uncertainty about the effect of Covid on mother and foetus, or simply general uncertainty at the onset and early stages of the pandemic. It is also possible, though unlikely given the age profile of infection and mortality in Covid, that fertility was adversely affected via direct exposure to the virus. In this case, while cumulative excess mortality may be negative this does not arise for any of the reasons discussed in section 2.

Table 1 shows rank correlations for cumulative excess mortality by age group and for the overall population. Consistent with the difficulties discussed above in interpreting the 0–14 figures, we see no rank correlation between excess mortality for this group and any other group. For other age groups the correlations are all positive and highly significant, though it is noticeable that the correlation between the over 85 age groups and younger groups is less than the inter-group correlations for the 15–64, 64–75 and 75–84 age groups. It is outside the scope of this study to investigate the reasons behind this result but it seems plausible that this may reflect differing experiences with respect to mortality rates in nursing homes.

**Table 1:** Spearman Correlations between age-specific mortality rates.

	Total	0-14	15-64	65-74	75-84	>85
**Total**	1.000					
**0-14**	0.128	1.000				
**15-64**	0.911***	0.205	1.000			
**65-74**	0.894***	0.146	0.862***	1.000		
**75-84**	0.867***	−0.082	0.834***	0.721***	1.000	
**>85**	0.616***	−0.020	0.470***	0.631***	0.497***	1.000

### Years of life lost (YLL)

We nowapply the GBD life table to calculate YLL for the countries in our sample and examine how the rankings by country differ from the rankings based simply on excess mortality (both unadjusted and age adjusted). If deaths are higher in younger age categories for any given country, then even controlling for the age distribution, this will give higher YLL, relative to where deaths predominantly occur in an older age category.

The GBD life table is reproduced in Table 2 for the relevant end-points of our age categories. Note that as we choose the end point of each age category our figures are a lower bound. Take, for example, the 15–64 age group. Life expectancy ranges here from 74 years for a 15 year old to 25.7 for a 65 year old (the GBD life table is in 5 year intervals, so we do not have precise figures for 64, 74, 84 year olds etc). While it seems likely that within that 15–64 year age category most of the excess mortality would have been towards the end of that category, at least some would have been amongst those in their 20s, 30s, 40s and 50s, whose YLL would have been higher. Hence, applying a YLL of 25.7 on *all* excess mortality in that age interval is imposing a lower bound. However, since information on the distribution of excess mortality *within* our age categories is lacking, we must employ a uniform value of YLL for each category. Our choice of the end-point is the most conservative choice. For our age category of over 85, we choose the YLL for 90 from the GBD life table. It is important to note that the use of the GBD life table for all countries does impose a degree of standardisation on the data. Almost certainly life expectancy, say at age 65, will differ by country. The GBD life table is based on the lowest observed age specific mortality rates for different age groups for the 204 countries in the GBD study.

**Table 2:** GBD Life Table.

Age	Life Expectancy
15	74.07
65	25.68
75	17.10
85	9.99
>85	7.62

Thus, for each country we multiply excess deaths in that age category by the corresponding YLL to obtain YLL for each category, which we then aggregate to obtain overall YLL. However, we need to express this relative to some underlying population. We construct this relevant population by simply multiplying the numbers in each age category for each country by the corresponding YLL to obtain what we term potential YLL (PYLL). Actual YLL is then expressed relative to that PYLL.

We also calculate YLL with a further age adjustment by applying the Eurostat age distribution to the YLL for each category and also using this age distribution to calculate PYLL. This measure calculates what total YLL would have been with the age-specific YLL numbers adjusted to the Eurostat age distribution. So, if YLL for age category 15–64 for country A was, say, 300000, and this category was, say 62% of population in country A as opposed to 64% for the Eurostat age distribution, then we multiply the 300000 by 1.032 (=.64/.62). Applying this to all age categories we have an adjusted total YLL which is expressed relative to adjusted PYLL.

Table 2 in supplementary file S1 Tables in S1 File shows the rankings of countries from high to low in terms of these mortality metrics, while Table 3 provides the Spearman rank correlations. Simple eye-balling of Table S2 in S1 File suggests that the rankings are very similar and this is confirmed by the correlations in Table 3, all of which are above 0.95 and highly significant (the relevant scatter plots are also presented in S2 Figures in S2 File). The message is fairly clear. No matter which of these mortality metrics is used, the rankings are very similar.

**Table 3:** Spearman Correlations Mortality Metrics.

	Excess Mortality	Age Adjusted Excess Mortality	YLL	Age adjusted YLL
**Excess Mortality**	1.000			
**Age Adjusted Excess Mortality**	0.9561***	1.000		
**YLL**	0.9835***	0.9567***	1.000	
**Age adjusted YLL**	0.9597***	0.9723***	0.9802***	1.000

### Relationship between excess mortality and GDP per capita and gini coefficient

**Fig 6:**
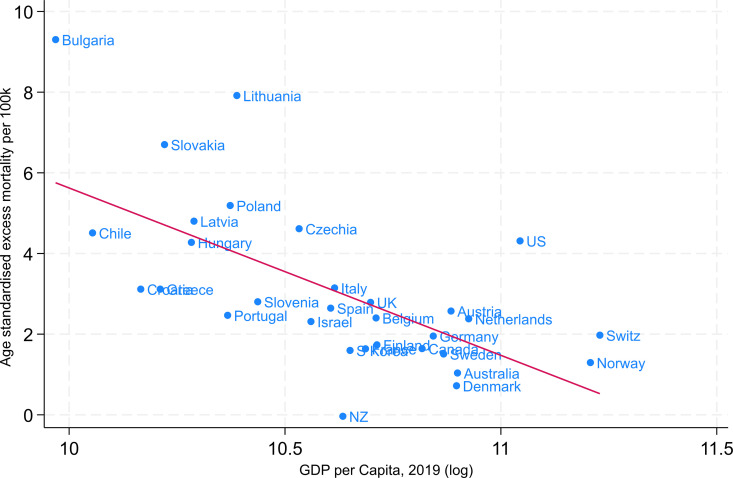
Age-Standardised Excess Mortality versus Log GDP per capita (linear fit).

**Fig 7:**
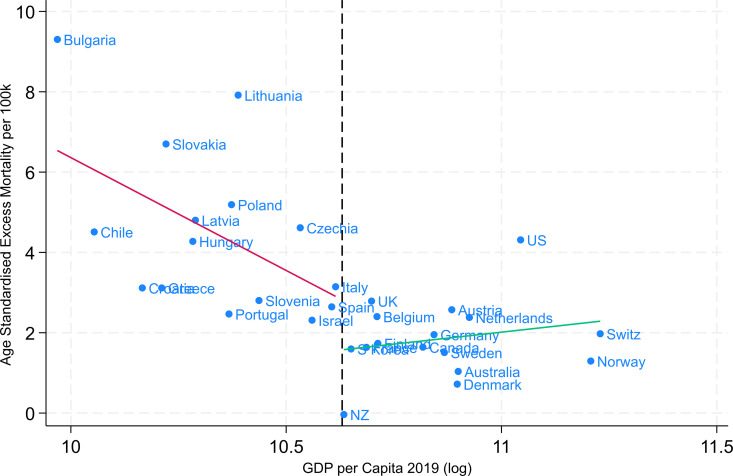
Age-Standardised Excess Mortality versus Log GDP per capita (linear spline).

**Fig 8:**
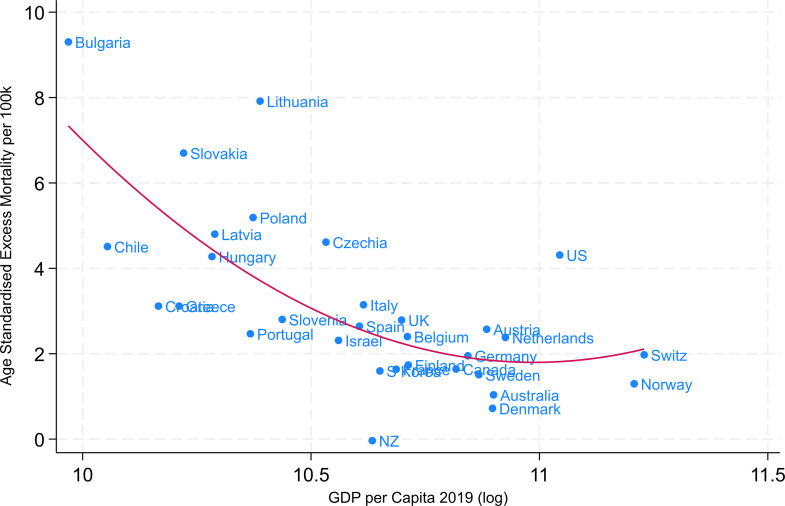
Age-Standardised Excess Mortality versus Log GDP per capita (quadratic fit).

Figs 6–8 present scatter plots for age adjusted excess mortality and the log of GDP per capita as evaluated in 2019, just before the start of Covid. To conserve space, and since there is such a strong correlation between the different mortality metrics, we just present results using age adjusted excess mortality. GDP per capita figures are obtained from the Penn World tables as provided by Our World in Data and are expressed in international dollars at 2017 prices. See https://archive.ourworldindata.org/20250805-101640/grapher/gdp-per-capita-penn-world-table.html?tab=table. We also show the “line of best fit” for three different specifications: the first is linear, the second is a linear spline with the knot at the median log GDP per capita value of 10.75, while the final is a quadratic. Fig 6 shows the linear fit, which shows a clear, negative, relationship between GDP per capita and excess mortality.

Eye-balling Fig 6 the relationship looks as though it may be flattening out as countries get richer and so Figs 7 and 8 show alternative specifications. In Fig 7 we have a linear spline with the knot at median GDP per capita (New Zealand). The negative relationship for countries with below median GDP per capita remains and it now looks as though there is a slight positive relationship for the richer countries. Fig 8 is the quadratic specification and is qualitatively very similar to the linear spline.

**Fig 9:**
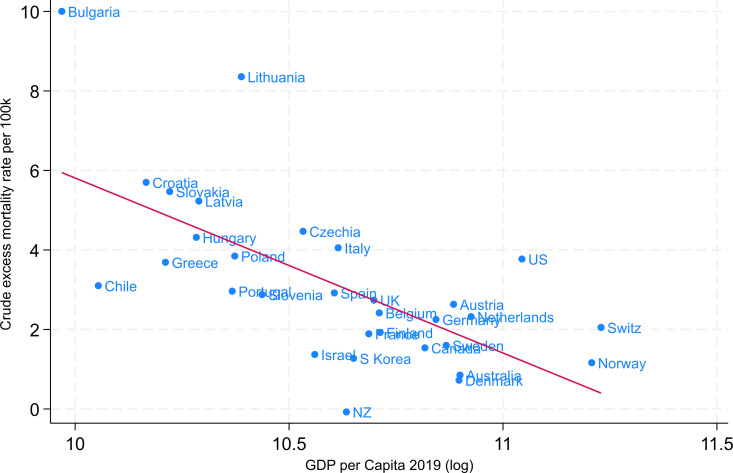
Crude Excess Mortality versus Log GDP per capita (linear fit).

The importance of adjusting for age can be seen by comparing Fig 6 and Fig 9. In Fig 9 we reproduce the linear fit scatter plot of Fig 6 but this time for crude excess mortality. The relative performance of countries such as Italy, Croatia and Slovakia changes quite significantly when the age adjustment is made, favourably in the case of Italy and Croatia but unfavourably for Slovakia.

**Fig 10:**
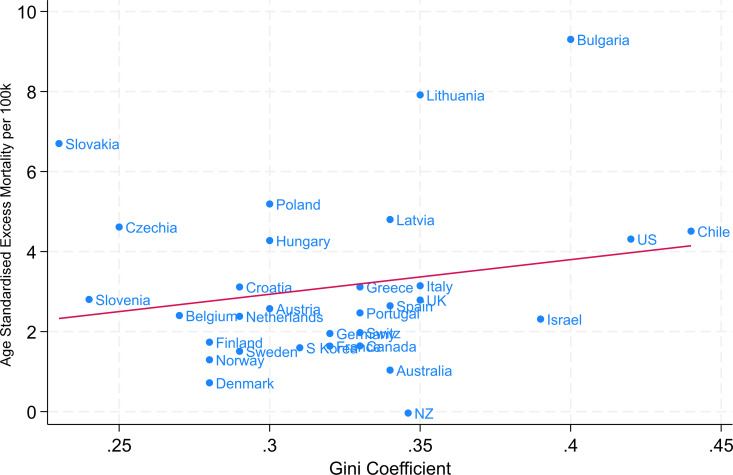
Age-Standardised Excess Mortality versus Gini Coefficient.

Finally, in Fig 10, we show the relationship between age standardised excess mortality and inequality as measured by the Gini coefficient for after tax disposable income (for 2019, as provided by Our World in Data). Again, we show the best fit linear regression which appears to indicate a positive relationship between excess mortality and inequality. However this slope is not statistically significant with a p-value of 0.27.

### Cluster analysis

Table 4 gives the values for the stopping rules for the Calinski_Harabasz, Duda-Hart and Pseudo T squared measures discussedabove. Note we present results for excess mortality-GDP per capita and excess mortality-Gini but we do not apply cluster analysis to the three variables together, excess mortality, GDP per capita and the Gini coefficient, simultaneously. This is because our interest lies in trying to identify clusters with respect to excess mortality and the two other variables of interest, GDP per capita and the Gini. Applying the analysis to all three variables simultaneously would also incorporate the relationship between GDP per capita and the Gini which, while an interesting research question in its own right, is not of concern to us here.

**Table 4:** Hierarchical Cluster Analysis – Stopping Rule Values.

Clusters	Age Standardised Excess Mortality and GDP per Capita			Age Standardised Excess Mortality and Gini		
	Calinski Harabasz	Duda Hart	Pseudo T Squared	Calinski Harabasz	Duda Hart	Pseudo T Squared
**2**	19.12	0.4004	38.93	12.22	0.6432	14.98
**3**	40.29	0.5792	12.35	16.64	0.6257	14.35
**4**	39.51	0.1863	4.37	21.18	0.6667	10.5
**5**	33.36	0.0411	23.34	23.02	0.2172	3.6
**6**	30.41	0.7713	4.15	20.53	0.8341	3.58
**7**	28.35	0.5166	6.55	19.33	0.0724	12.8

**Fig 11:**
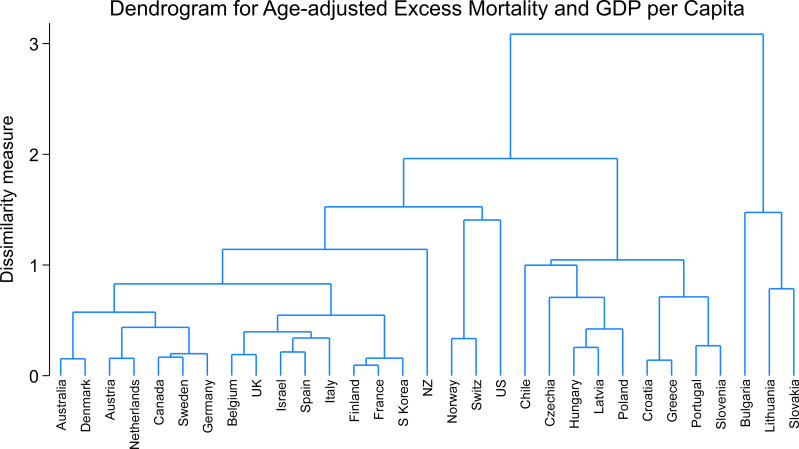
Dendrogram for Age Adjusted Excess Mortality and GDP per Capita.

**Fig 12:**
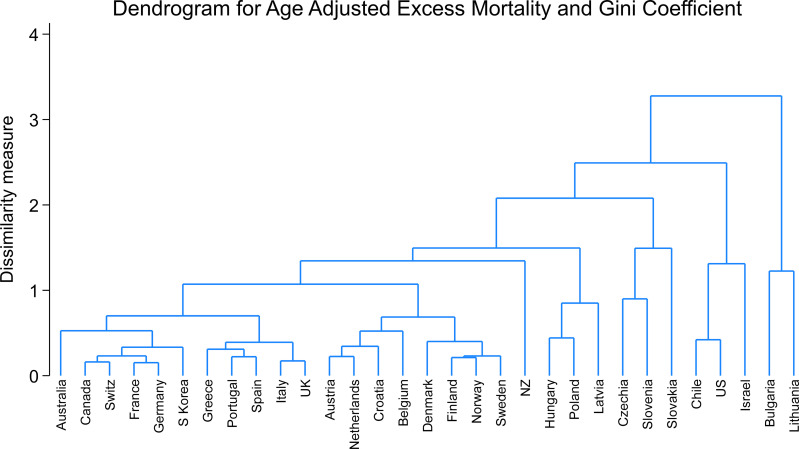
Dendrogram for Age Adjusted Excess Mortality and Gini Coefficient.

The results in Table 4 suggest possible clusters of 3 and 4 for excess mortality-GDP per capita and 4 and 5 for excess mortality-Gini. We can also observe this in the respective dendrograms in Figs 11 and 12. Visual inspection of these dendrograms indicate optimal values of 3 and 4 respectively, since this is where the vertical gaps in the dissimilarity measure are greatest, and Table 3 and 44 (in supplementary file S1 Tables in S1 File) give the clusters by country. Checking back to Fig 6, for the excess mortality-GDP clusters, we see that Bulgaria, Lithuania and Slovakia clearly stand out as countries with low GDP per capita and high excess mortality. The next cluster of nine countries again feature those with lower excess mortality and relatively higher GDP per capita but still below median GDP per capita. The remainder consist of countries with either quite low excess mortality and/or GDP per capita around or above the median. This final cluster can include countries with quite diverse experiences, e.g., New Zealand has very low excess mortality but median GDP per capita, whereas the US has quite high excess mortality but GDP per capita well above the median.

Finally, Fig 12 shows the scatter plot for clustering with the Gini coefficient. The cluster analysis identifies Bulgaria and Lithuania as an outlying group with very high excess mortality and high or medium levels of inequality. A second cluster comprises Israel, Chile and the US, three countries with lower excess mortality but high levels of inequality. There is then a three-group cluster of Czechia, Slovakia and Slovenia, all with very low inequality, though varying levels of excess mortality. The final cluster, comprising 23 countries are effectively a central “bloc” in the scatter diagram with a relatively narrow range of both excess mortality and inequality.

## Discussion

This paper analysed excess mortality over the “Covid period” of January 2020-December 2022 for a selection of countries, the choice of which was determined by data availability. The measure of excess mortality employed, that of Karlinsky and Kobak, was shown to be close to other available measures of excess mortality, excepting the IHME data which appears to be a clear outlier. The paper also applied age standardization to the excess mortality series to take account of differing age structures by country. The effect of such age standardisation is perhaps most prominent in the case of Italy. Its crude excess mortality is just over 4 per one hundred thousand. However, if it had the standard Eurostat age structure, *and assuming its age specific excess mortality had been the same*, then excess mortality would have been about a quarter lower. The underlying assumption, highlighted in italics, is critically important. Age-specific mortality rates might not be independent of age structure. For example, if a country has a high fraction of its population, say, over the age of 75, then medical resources and facilities for this age group might be of very high quality, which in turn would presumably bring *down* age specific mortality for that group. This is potentially an important issue, but one beyond the scope of this paper to address.

Of course, age-structure could also reduce crude excess mortality to below what it would have been if a country had the Eurostat standard. For example, Chile recorded crude excess mortality of 3.1 per one hundred thousand, but if its age structure had been the Eurostat standard then its age-specific excess mortality would have resulted in crude excess mortality almost 50 per cent higher at around 4.5. Overall, however, the ranking of countries by excess mortality changed little with the standardization.

Further analysis investigated whether the rankings differed according to age bracket. Dealing first of all with the very youngest age-bracket, previous pandemics, such as the 1918 H1N1 Flu, and the more recent Ebola and Zika outbreaks have been associated with declines in birth rates nine months after their peaks [25]. Pomar et al [25] investigate the effect of the first wave of Covid on births in 24 European countries, by looking at live births in January 2021 (approximately nine months after the peak of wave 1 of Covid) compared to the average of January 2018−19 and taking account of secular trends and seasonality. They find a drop of over 14 per cent, though there is a partial rebound in March 2021. They find no change in the trend of live births in the early months of the pandemic, suggesting little effect arising from direct exposure to Covid. They also find that the reduction in births is greater where medical systems were under strain (as measured by ICU occupancy) and also by duration and severity of lockdown, which in turn of course is likely to be affected by medical system strain. Adelman et al [26] carried out a similar study for the US, finding a decline in fertility for wave 1 of Covid but this was not replicated in subsequent waves.

On balance, it seems that fertility (and consequently perinatal mortality) was influenced by Covid, but not uniformly by wave. It is possible that fertility initially declined but then rebounded, so that it is difficult to discern an effect on cumulative mortality. We checked how excess mortality for the 0–14 age group varied over 2020–2022. Since Covid cases for the vast majority of countries were not recorded before March 2020, for planned pregnancies before that date and hence for births up to around the end of 2020, Covid should not have been a factor in these decisions. Hence, we would expect excess mortality for 2020 to exceed that for 2021. Yet that is not the case. Only in eight of our selected countries is excess mortality in 2020 higher than in 2021.

So, while there is evidence of a Covid effect on fertility it may simply be too slight to be picked up in our excess mortality figures, figures which of course apply to a much wider age range than that for perinatal mortality. Nevertheless, these factors should be borne in mind when interpreting results by age group.

Leaving aside those in the 0–14 age group, where most mortality happens in the first six months of life, again there was a very high correlation in the ranking of countries by age-specific excess mortality.

The very high rank correlation also extended to the years of life lost metric, confirming that country rankings of excess mortality, for this selection of countries at least, is highly robust to choice of mortality metric. This result is important, as it is possible that debates about relative country performances during the Covid pandemic could become confused owing to the use of different metrics. This analysis suggests that, for this set of countries at least, relative performance is very little affected by choice of mortality metric.

The final part of the paper investigated the relationship between mortality metrics and two key population-level indicators of economic performance, GDP per capita and income inequality. This analysis was extended by applying hierarchical cluster analysis to investigate if any patterns could be found regarding groups of countries in terms of excess mortality, GDP per capita and the Gini coefficient. It found that in general there was an inverse relationship between excess mortality and GDP per capita, but there are the outliers in this relationship. Bulgaria, Lithuania, Slovakia and the US all underperform in the sense of having higher excess mortality than would be predicted by their GDP per capita. On the other side New Zealand is a clear outlier but Croatia, Greece and Portugal also do “better” than their GDP per capita would predict.. Closer analysis of the linear relationship suggests it may be flatten out as countries get richer. Trying to take account of this by introducing a spline at median GDP per capita shows that the negative relationship for countries with below median GDP per capita remains and it now looks as though there is a slight positive relationship for the richer countries. However this is very much driven by the outlier which is the US. For other countries with above median GDP per capita there is effectively no relationship between excess mortality and GDP per capita. A quadratic specification is qualitatively very similar to the linear spline. Apart from the US, the relationship between excess mortality and GDP per capita is weak for the richer countries.

The comparison of Figs 6 and 9 show the importance of the age adjustment Fig 9 reproduces the linear fit scatter plot of Fig 6 but this time for crude excess mortality. The relative performance of countries such as Italy, Croatia and Slovakia is sensitive to the age adjustment. The relative performances of Italy and Croatia improve, while that of Slovakia is worse..

A weakly positive, though not statistically significant, relationship is observed between excess mortality and the Gini coefficient.It is noticeable however, that countries above the regression line and who could be viewed as having a worse excess mortality outcome given their Gini coefficient are former centrally planned east European countries. This raises the possibility that there may be clusters of countries with similar outcomes for excess mortality and GDP per capita/inequality and this can be analysed more formally using hierarchical cluster analysis.

The cluster analysis suggests that some of the former communist countries of Europe had similar experiences with respect to high rates of excess mortality and low GDP per capita. In terms of excess mortality and the Gini coefficient the picture was more nuanced, with these countries, all of whom had high excess mortality, now further broken down into groups with high and low Gini coefficients. The other groups produced by the cluster analysis were predominantly of relatively higher income advanced economies who had lower excess mortality and generally low Gini coefficients, with Israel, Chile and the US as exceptions.

## Conclusion

This paper investigates cross-country patterns in various metrics of excess mortality. As such, it acts as a useful precursor to possible supra-national examination of other Covid-related outcomes. The analysis shows that rankings across countries are very robust to age adjustment and alternative mortality metrics. The cluster analysis also aids in identifying which countries are “different” and which countries are “similar”. As a final caveat however, it must be borne in mind that the sample of countries analysed in this paper was driven by data considerations and the sample should not be regarded as “representative”. Thus caution is advised in terms of extrapolating results from this analysis to other countries. Nevertheless, the paper offers a valuable starting point for cross-country analysis of countries’ “performance” during the Covid pandemic and hopefully provides insights for future worldwide health shocks.

## Supporting information

S1 File Table 1: Rankings by age-specific mortality rates. Table 2: Rankings by Different Mortality Metrics. Table 3: Cluster Membership, Age Standardised Excess Mortality and GDP per Capita. Table 4: Cluster Membership, Age Standardised Excess Mortality and Gini Coefficient.
(PDF)

S2 File Figure 1: Raw excess mortality rank versus age adjusted excess mortality rank. Figure 2: Raw excess mortality versus raw years of life lost. Figure 3: Raw excess mortality versus age adjusted years of life lost. Figure 4: Age adjusted excess mortality versus raw years of life lost. Figure 5: Age adjusted excess mortality versus age adjusted raw years of life lost. Figure 6: Raw years of life lost versus age adjusted raw years of life lost.
(PDF)
