# Supplementary material for: Covid-19 related excess mortality: An analysis by age for selected countries
Source: PLoS One. 2026 Jul 16;21(7):e0353766. doi: 10.1371/journal.pone.0353766 (PMC13374911; doi:10.1371/journal.pone.0353766)
Supplement: S1 File — Table 2: Rankings by Different Mortality Metrics. Table 3: Cluster Membership, Age Standardised Excess Mortality and GDP per Capita. Table 4: Cluster Membership, Age Standardised Excess Mortality and Gini Coefficient. (PDF) [file pone.0353766.s001.pdf]

Table 1: Rankings by Age-specific Mortality Rates

0-14	15-64	65-74	75-84	>84	Total
Canada	Lithuania	Bulgaria	Bulgaria	Bulgaria	Bulgaria
Lithuania	Bulgaria	Lithuania	Slovakia	Lithuania	Lithuania
Latvia	Latvia	Latvia	Czechia	Croatia	Croatia
Switz	US	Croatia	Poland	Slovakia	Slovakia
Croatia	Slovakia	Slovakia	Lithuania	Poland	Latvia
Hungary	Hungary	Hungary	Chile	Chile	Czechia
Sweden	Chile	Poland	Croatia	Germany	Hungary
US	Czechia	US	Hungary	Italy	Italy
Denmark	Poland	Chile	Belgium	Israel	Poland
Spain	Croatia	Germany	US	Switz	US
UK	Greece	Czechia	Greece	S Korea	Greece
S Korea	Canada	Austria	Netherlands	UK	Chile
Netherlands	UK	Italy	Spain	Portugal	Portugal
Greece	Slovenia	Slovenia	Italy	Greece	Spain
Slovakia	Italy	S Korea	Latvia	Czechia	Slovenia
France	Belgium	Greece	UK	Slovenia	UK
Germany	Austria	Spain	Austria	US	Austria
Austria	Spain	Portugal	Slovenia	Spain	Belgium
Italy	Portugal	UK	Israel	Netherlands	Netherlands
Bulgaria	Germany	Belgium	Portugal	Latvia	Germany
Norway	Netherlands	Netherlands	Finland	Finland	Switz
Israel	Finland	Canada	France	France	Finland
Finland	Sweden	Israel	Sweden	Sweden	France
Australia	Switz	Switz	Norway	Norway	Sweden
Slovenia	Israel	France	Canada	Belgium	Canada
Poland	France	Australia	Switz	Austria	Israel
NZ	Norway	Finland	Australia	Australia	S Korea
Portugal	Denmark	NZ	Denmark	Hungary	Norway
Czechia	S Korea	Sweden	NZ	Denmark	Australia
Chile	Australia	Norway	S Korea	Canada	Denmark
Belgium	NZ	Denmark	Germany	NZ	NZ

Table 2: Rankings by Different Mortality Metrics

Excess Mortality	Age Adjusted Excess Mortality	YLL	Age adjusted YLL
Bulgaria	Bulgaria	Bulgaria	Bulgaria
Lithuania	Lithuania	Lithuania	Lithuania
Croatia	Slovakia	Latvia	Slovakia
Slovakia	Poland	Croatia	Latvia
Latvia	Latvia	Slovakia	Croatia
Czechia	Czechia	Hungary	US
Hungary	Chile	Poland	Poland
Italy	US	US	Hungary
Poland	Hungary	Czechia	Chile
US	Italy	Italy	Czechia
Greece	Croatia	Greece	Greece
Chile	Greece	Chile	Italy
Portugal	Slovenia	Slovenia	Slovenia
Spain	UK	Austria	UK
Slovenia	Spain	Spain	Austria
UK	Austria	Portugal	Spain
Austria	Portugal	UK	Portugal
Belgium	Belgium	Germany	Canada
Netherlands	Netherlands	Netherlands	Belgium
Germany	Israel	Belgium	Netherlands
Switzerland	Switzerland	Canada	Germany
Finland	Germany	Switzerland	Israel
France	Finland	Finland	Switzerland
Sweden	Canada	France	Finland
Canada	France	Sweden	S Korea
Israel	S Korea	S Korea	Sweden
S Korea	Sweden	Israel	France
Norway	Norway	Norway	Norway
Australia	Australia	Australia	Australia
Denmark	Denmark	Denmark	Denmark
NZ	NZ	NZ	NZ

Table 3: Cluster Membership, Age Standardised Excess Mortality and GDP per Capita

Cluster	Countries
**1**	Australia, Austria, Belgium, Canada, Finland, France, Germany, Israel, Italy, New Zealand, Netherlands, Norway, South Korea, Spain, Sweden, Switzerland, UK, US
**2**	Chile, Croatia, Czechia, Greece, Hungary, Latvia, Poland, Portugal, Slovenia
**3**	Bulgaria, Lithuania, Slovakia

Table 4: Cluster Membership, Age Standardised Excess Mortality and Gini Coefficient

Cluster	Countries
**1**	Australia, Austria, Belgium, Canada, Croatia, Finland, France, Germany, Greece, Hungary, Italy, Latvia, New Zealand, Netherlands, Norway, Poland, Portugal, South Korea, Spain, Sweden, Switzerland, UK
**2**	Czechia, Slovakia, Slovenia
**3**	US, Israel, Chile
**4**	Bulgaria, Lithuania
