# Supplementary material for: Covid-19 related excess mortality: An analysis by age for selected countries
Source: PLoS One. 2026 Jul 16;21(7):e0353766. doi: 10.1371/journal.pone.0353766 (PMC13374911; doi:10.1371/journal.pone.0353766)

Figure 1: Raw excess mortality rank versus age adjusted excess mortality rank

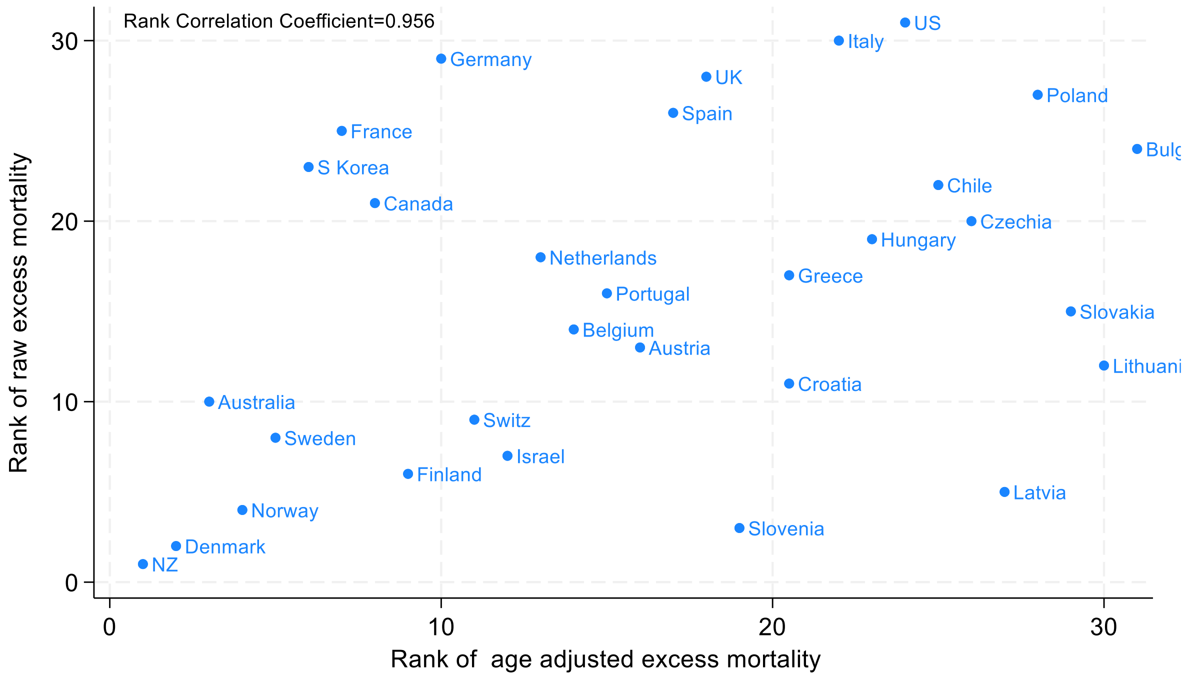

Figure 2: Raw excess mortality versus raw years of life lost

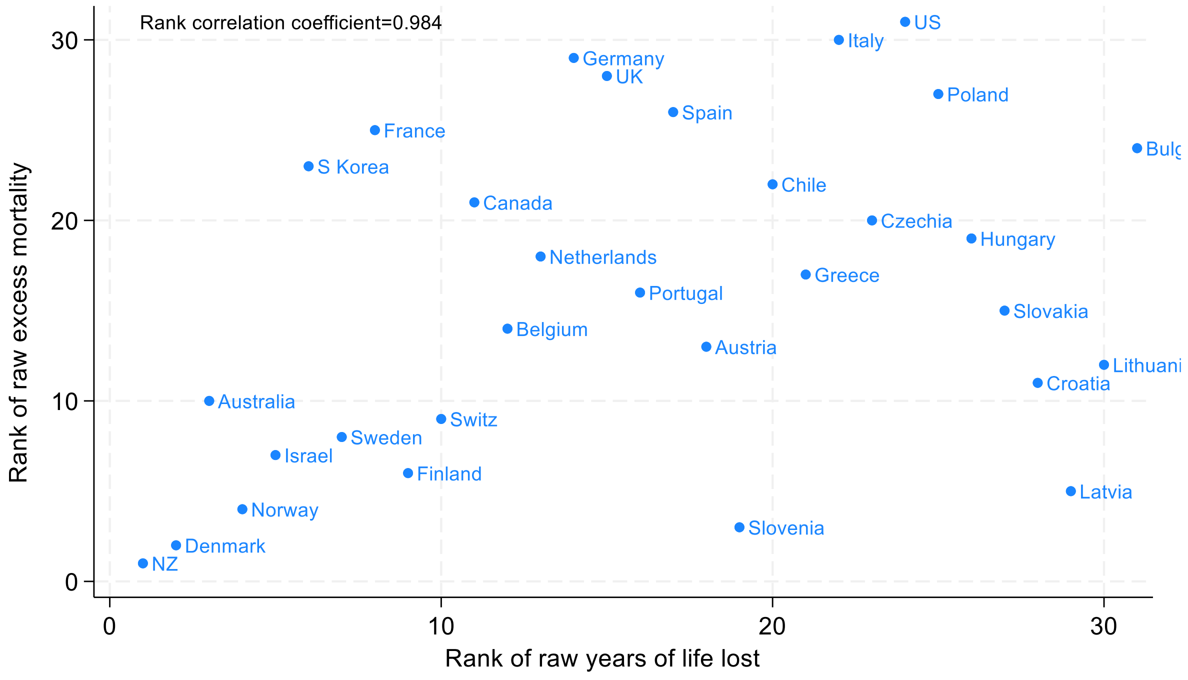

Figure 3: Raw excess mortality versus age adjusted years of life lost

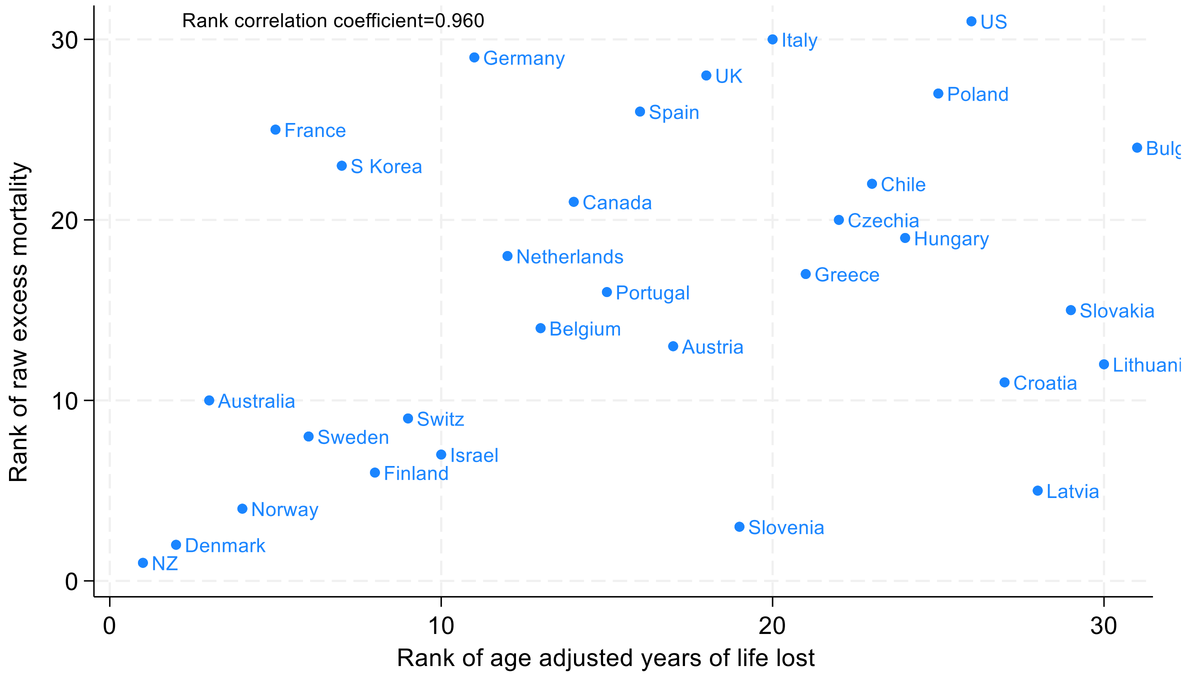

Figure 4: Age adjusted excess mortality versus raw years of life lost

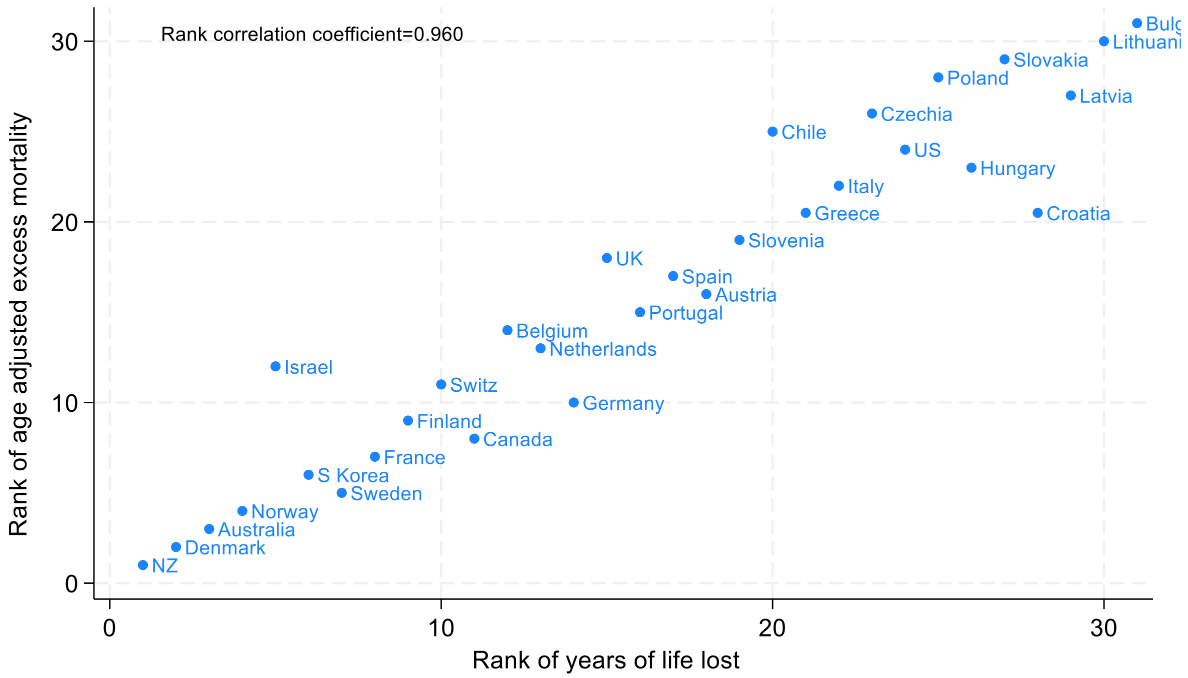

Figure 5: Age adjusted excess mortality versus age adjusted raw years of life lost

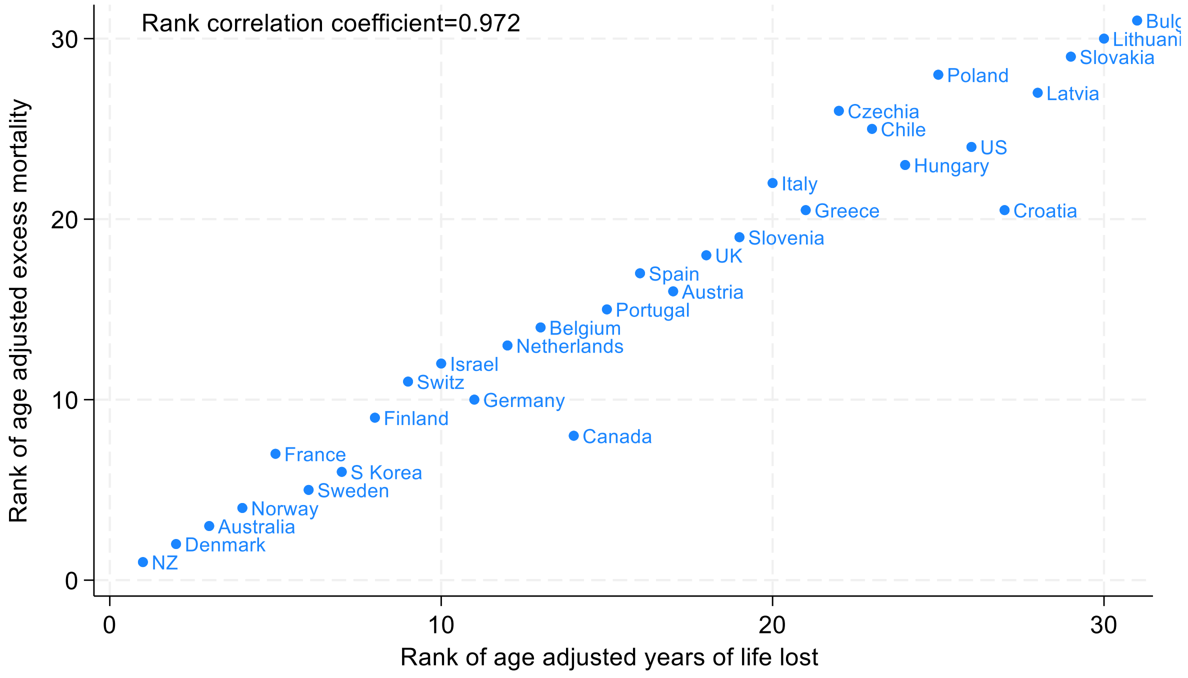

Figure 6: Raw years of life lost versus age adjusted raw years of life lost

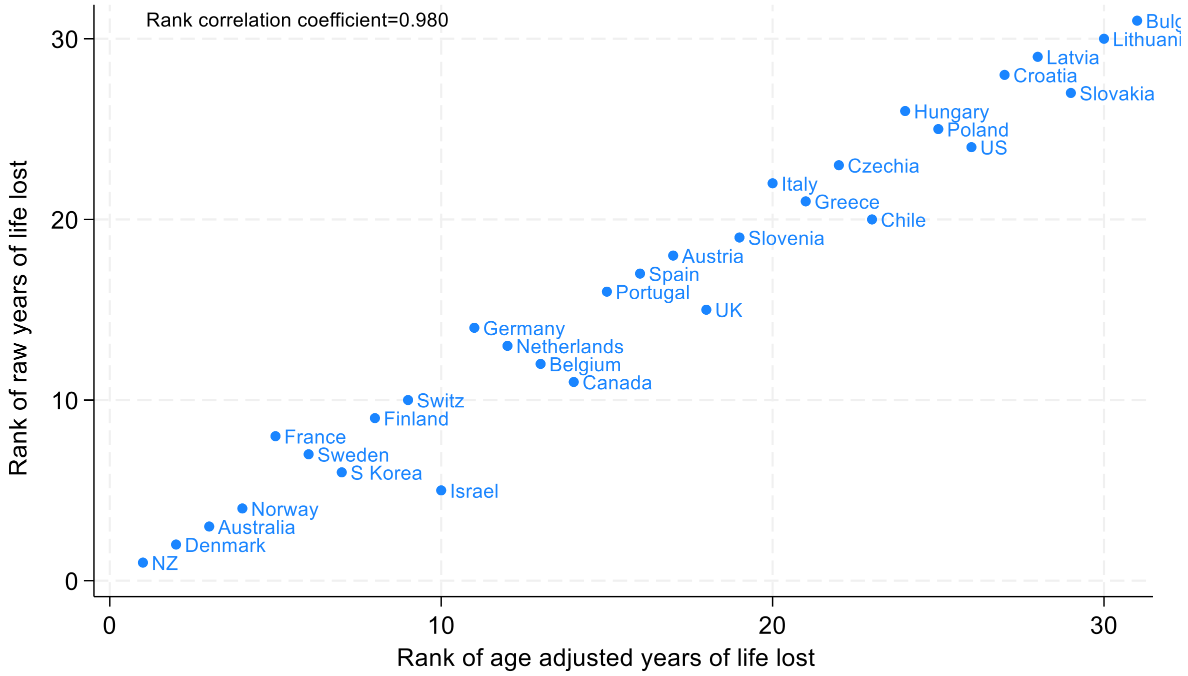

Supplement: S2 File — Figure 2: Raw excess mortality versus raw years of life lost. Figure 3: Raw excess mortality versus age adjusted years of life lost. Figure 4: Age adjusted excess mortality versus raw years of life lost. Figure 5: Age adjusted excess mortality versus age adjusted raw years of life lost. Figure 6: Raw years of life lost versus age adjusted raw years of life lost. (PDF) [file pone.0353766.s002.pdf]
