# Supplementary material for: Covid-19 related excess mortality: An analysis by age for selected countries
Source: PLoS One. 2026 Jul 16;21(7):e0353766. doi: 10.1371/journal.pone.0353766 (PMC13374911; doi:10.1371/journal.pone.0353766)
Supplement: Attachment — Submitted filename: Response to Reviewers.docx [file pone.0353766.s004.docx]

To the reviewers and editor: Thank you very much for taking the time to read my paper. I will do my best to respond to all the issues raised. Below I provide my responses on a point by point basis.

**Reviewer #1: .**

**1. The abstract in its current form is not sufficiently structured and lacks key quantitative details. While it outlines the general analytical approach, it does not provide specific numerical results (e.g., correlation coefficients, effect sizes, or statistical significance levels), which limits its scientific clarity and impact. A more structured abstract (background, methods, results, conclusions) with clearly reported numerical findings is strongly recommended.**

Thank you for this comment. The word limit for the abstract is 300 words, which is why my original abstract was quite concise. In the revised version I have now structured the abstract more formally, using a background/methods/results/conclusions approach. I have also included some numerical results concerning rank correlation coefficients across the different mortality metrics and also with respect to GDP per capita and the Gini coefficient. The revised abstract is still within the 300 word limit.

**2. More broadly, the manuscript is difficult to follow due to the lack of adherence to a clear IMRAD structure. The introduction does not adequately establish the relevance and novelty of the study. It lacks a clear articulation of the research gap and does not sufficiently reference key prior studies in the field of excess mortality during COVID-19. Strengthening the justification of the study and situating it within existing literature would significantly improve the manuscript.**

Thank you for this comment. The original manuscript adhered loosely to the “Manuscript Organization” outline in the PLOS submission guidelines. I have restructured the manuscript more strictly according to the IMRAD norms.

In response to the second part of this comment, I have reviewed a number of key prior studies and placed my own study in the context of their contribution. I point out that different studies have examined the importance of (a) different estimates of excess mortality (b) different metrics such as years of life lost and (c) the impact of age adjustment. However, to the best of my knowledge there is no paper which addresses all of these issues simultaneously, and my paper fills this gap. In addition, I included other population level factors such as GDP per head and country inequality in my analysis.

**3. The methodology section requires clearer and more structured description. In particular, the rationale for selecting the specific dataset and the included countries is not sufficiently explained. It would be important to clarify why these countries were chosen, what makes them comparable or analytically meaningful, and how this selection supports the study objectives and potential generalizability of findings.**

Thank you for this comment. In line with the revision to the structure of the paper according to IMRAD norms, I have restructured the methodology section and hopefully it is now clearer. In terms of the choice of measure and countries, there are a number of estimates of excess mortality available and which particular one to choose is very important. Hence I devote some time to this. On balance the K&K choice seemed best. Critically It has estimates of excess mortality by age group, which enables the age adjustment to be made. The choice of countries was dictated by those countries for which the breakdown of excess mortality by age was available and hence this is not a “representative” sample of countries, and I have pointed out that other studies in the field have had to take this approach. I have inserted a number of statements to make it absolutely clear that this sample is thus not in any way “representative”. I make clear in the conclusions section that the extrapolation of results from this sample is also subject to this caveat.

**4. The presentation of results could also be improved. Correlation analyses would be more effectively communicated through graphical representations rather than relying predominantly on tables. Some tables appear overly dense and reduce readability; in particular, tables with country rankings could be moved to supplementary materials.**

Thank you for this comment. I have removed four tables to supplementary materials *S1 Tables*: the country rankings for age-specific rankings (originally table 1), the rankings by different mortality metrics (originally table 4) and the two tables listing the clusterings (originally tables 7 and 8). I thought it best not to replace all of these tables with a graph. However, I have inserted six extra graphs into the supplementary material in file *S2 Figures* which provides the material from the original table 4 in graphical form (I am happy to put these graphs into the main text if requested by referee/editor).

**5. Finally, the conclusions should be more concise and clearly linked to the main findings, with a stronger emphasis on their implications.**

Thank you. I have rebalanced the Discussion and Conclusion sections. The Conclusion section is now much shorter, and summarises the findings, enters the caveat regarding the sample of countries analysed and stresses that the paper has value in terms of future studies which may examine Covid-related outcomes.

**Reviewer #2:**

**1. It has scientific merit, however some clarifications should be made for general audiences who can not be familiar with the technical nature of the topic. For example, added value with this study should be obviously explained in the discussion. You should give clear explanations on why your findings differed from various studies and strengths, and drawbacks with your study design. This revisions will lead your discussion part getting confident with scope.**

Thank you for this comment. This comment overlaps significantly with comment 2 of reviewer 1, hence I refer to my response to that comment. In terms of drawbacks of this study, I have added emphasis on the narrow range of countries included (by data necessity) and stressed that extrapolation of results here to other countries should only be done with great care.

**2. Additionally, there exist plenty of sentences in findings section that can easily go to the discussion, so your own findings should remain in their places. Lastly, during the covid pandemic period, both local studies and comparative studies were published to evaluate excess mortality, so you may consider to discuss your results with their excess mortality data.**

Thank you for this comment. I have re-balanced the paper, and moved some text from the “Results” section to the “Discussion” section.

In response to the second part of this comment, I have expanded the Introduction section and within that I have included reviews of some of the most relevant comparative studies and compared their approach to mine.

**Additional editor comments**
