# Supplementary material for: Covid-19 related excess mortality: An analysis by age for selected countries
Source: PLoS One. 2026 Jul 16;21(7):e0353766. doi: 10.1371/journal.pone.0353766 (PMC13374911; doi:10.1371/journal.pone.0353766)
Supplement: Peer review history

# Peer review: Decision Letter 0

**DOI:** 10.1371/journal.pone.0353766.r001

**Authors:** Muhammad Umair

9 Apr 2026

PONE-D-26-08812

Covid-19 Related Excess Mortality: An Analysis by Age for Selected Countries

PLOS One

Dear Dr. Madden,

Thank you for submitting your manuscript to PLOS ONE. After careful consideration, we feel that it has merit but does not fully meet PLOS ONE’s publication criteria as it currently stands. Therefore, we invite you to submit a revised version of the manuscript that addresses the points raised during the review process.

Both reviewers acknowledge the scientific merit of the study and the robustness of the statistical analyses. However, they have identified several areas that require substantial improvement. The abstract should be restructured to include key quantitative results, such as correlation coefficients, effect sizes, and significance levels, and the manuscript would benefit from a more consistent IMRAD structure to improve clarity and readability. The introduction should more explicitly state the research gap, novelty, and relevance of the study, with additional references to prior studies on COVID-19-related excess mortality and age-specific analyses.

The methodology section requires clearer articulation of the rationale for selecting specific datasets and countries, along with detailed explanations of data sources, comparability, and the generalizability of the findings. In terms of results presentation, certain tables are dense and would benefit from graphical representations to enhance interpretability, and some descriptive or interpretive content in the results section should be moved to the discussion.

The discussion should clarify the added value of the study for general audiences, compare findings with existing local and international studies, and explicitly describe the strengths and limitations of the study design. The conclusions should be concise and directly linked to the main findings.

Overall, the reviewers agree that the study is relevant and potentially valuable, but substantial revisions are required to improve clarity, scientific presentation, and contextualization of results. I recommend that the authors be invited to revise the manuscript in accordance with the reviewers’ comments and resubmit for further evaluation. A detailed point-by-point response addressing each reviewer’s comment should accompany the revised submission.

Please submit your revised manuscript by May 24 2026 11:59PM. If you will need more time than this to complete your revisions, please reply to this message or contact the journal office at plosone@plos.org. When you're ready to submit your revision, log on to https://www.editorialmanager.com/pone/ and select the 'Submissions Needing Revision' folder to locate your manuscript file.

Please include the following items when submitting your revised manuscript:

- A letter that responds to each point raised by the academic editor and reviewer(s). You should upload this letter as a separate file labeled 'Response to Reviewers'.
- A marked-up copy of your manuscript that highlights changes made to the original version. You should upload this as a separate file labeled 'Revised Manuscript with Track Changes'.
- An unmarked version of your revised paper without tracked changes. You should upload this as a separate file labeled 'Manuscript'.

If you would like to make changes to your financial disclosure, please include your updated statement in your cover letter. Guidelines for resubmitting your figure files are available below the reviewer comments at the end of this letter.

If applicable, we recommend that you deposit your laboratory protocols in protocols.io to enhance the reproducibility of your results. Protocols.io assigns your protocol its own identifier (DOI) so that it can be cited independently in the future. For instructions see: https://journals.plos.org/plosone/s/submission-guidelines#loc-laboratory-protocols. Additionally, PLOS ONE offers an option for publishing peer-reviewed Lab Protocol articles, which describe protocols hosted on protocols.io. Read more information on sharing protocols at https://plos.org/protocols?utm_medium=editorial-email&utm_source=authorletters&utm_campaign=protocols.

As the corresponding author, your ORCID iD is verified in the submission system and will appear in the published article. PLOS supports the use of ORCID, and we encourage all coauthors to register for an ORCID iD and use it as well. Please encourage your coauthors to verify their ORCID iD within the submission system before final acceptance, as unverified ORCID iDs will not appear in the published article. *Only*  the individual author can complete the verification step; PLOS staff *cannot*  verify ORCID iDs on behalf of authors.

We look forward to receiving your revised manuscript.

Kind regards,

Muhammad Umair, Ph.D.

Academic Editor

PLOS One

Journal Requirements:

When submitting your revision, we need you to address these additional requirements.

1. Please ensure that your manuscript meets PLOS ONE's style requirements, including those for file naming. The PLOS ONE style templates can be found at

https://journals.plos.org/plosone/s/file?id=wjVg/PLOSOne_formatting_sample_main_body.pdf and

https://journals.plos.org/plosone/s/file?id=ba62/PLOSOne_formatting_sample_title_authors_affiliations.pdf.

2. If the reviewer comments include a recommendation to cite specific previously published works, please review and evaluate these publications to determine whether they are relevant and should be cited. There is no requirement to cite these works unless the editor has indicated otherwise.

Additional Editor Comments (if provided):

I am writing regarding the manuscript entitled “Covid-19 Related Excess Mortality: An Analysis by Age for Selected Countries” (PONE-D-26-08812), submitted to PLOS ONE. After careful evaluation of the peer reviews, I recommend that the manuscript undergo major revision before it can be considered for acceptance.

Both reviewers acknowledge the scientific merit of the study and the robustness of the statistical analyses. However, they have identified several areas that require substantial improvement. The abstract should be restructured to include key quantitative results, such as correlation coefficients, effect sizes, and significance levels, and the manuscript would benefit from a more consistent IMRAD structure to improve clarity and readability. The introduction should more explicitly state the research gap, novelty, and relevance of the study, with additional references to prior studies on COVID-19-related excess mortality and age-specific analyses.

The methodology section requires clearer articulation of the rationale for selecting specific datasets and countries, along with detailed explanations of data sources, comparability, and the generalizability of the findings. In terms of results presentation, certain tables are dense and would benefit from graphical representations to enhance interpretability, and some descriptive or interpretive content in the results section should be moved to the discussion.

The discussion should clarify the added value of the study for general audiences, compare findings with existing local and international studies, and explicitly describe the strengths and limitations of the study design. The conclusions should be concise and directly linked to the main findings.

Overall, the reviewers agree that the study is relevant and potentially valuable, but substantial revisions are required to improve clarity, scientific presentation, and contextualization of results. I recommend that the authors be invited to revise the manuscript in accordance with the reviewers’ comments and resubmit for further evaluation. A detailed point-by-point response addressing each reviewer’s comment should accompany the revised submission.

Thank you for considering this recommendation. I remain available to provide further guidance as needed.

[Note: HTML markup is below. Please do not edit.]

Reviewers' comments:

Reviewer's Responses to Questions

**Comments to the Author**

1. Is the manuscript technically sound, and do the data support the conclusions?

The manuscript must describe a technically sound piece of scientific research with data that supports the conclusions. Experiments must have been conducted rigorously, with appropriate controls, replication, and sample sizes. The conclusions must be drawn appropriately based on the data presented.

Reviewer #1: Partly

Reviewer #2: Yes

**********

2. Has the statistical analysis been performed appropriately and rigorously?

Reviewer #1: Yes

Reviewer #2: Yes

**********

3. Have the authors made all data underlying the findings in their manuscript fully available?

The PLOS Data policy requires authors to make all data underlying the findings described in their manuscript fully available without restriction, with rare exception (please refer to the Data Availability Statement in the manuscript PDF file). The data should be provided as part of the manuscript or its supporting information, or deposited to a public repository. For example, in addition to summary statistics, the data points behind means, medians and variance measures should be available. If there are restrictions on publicly sharing data—e.g. participant privacy or use of data from a third party—those must be specified.

Reviewer #1: Yes

Reviewer #2: Yes

**********

4. Is the manuscript presented in an intelligible fashion and written in standard English?

PLOS ONE does not copyedit accepted manuscripts, so the language in submitted articles must be clear, correct, and unambiguous. Any typographical or grammatical errors should be corrected at revision, so please note any specific errors here.

Reviewer #1: Yes

Reviewer #2: Yes

**********

5. Review Comments to the Author

Please use the space provided to explain your answers to the questions above. You may also include additional comments for the author, including concerns about dual publication, research ethics, or publication ethics. (Please upload your review as an attachment if it exceeds 20,000 characters)

Reviewer #1: I read this manuscript with interest, as it addresses an important and timely topic COVID-19-related excess mortality across countries and age groups. The study raises relevant questions regarding the role of age standardization and socioeconomic factors in interpreting mortality patterns. However, several aspects of the manuscript, particularly the abstract and overall structure, require substantial improvement.

The abstract in its current form is not sufficiently structured and lacks key quantitative details. While it outlines the general analytical approach, it does not provide specific numerical results (e.g., correlation coefficients, effect sizes, or statistical significance levels), which limits its scientific clarity and impact. A more structured abstract (background, methods, results, conclusions) with clearly reported numerical findings is strongly recommended.

More broadly, the manuscript is difficult to follow due to the lack of adherence to a clear IMRAD structure. The introduction does not adequately establish the relevance and novelty of the study. It lacks a clear articulation of the research gap and does not sufficiently reference key prior studies in the field of excess mortality during COVID-19. Strengthening the justification of the study and situating it within existing literature would significantly improve the manuscript.

The methodology section requires clearer and more structured description. In particular, the rationale for selecting the specific dataset and the included countries is not sufficiently explained. It would be important to clarify why these countries were chosen, what makes them comparable or analytically meaningful, and how this selection supports the study objectives and potential generalizability of findings.

The presentation of results could also be improved. Correlation analyses would be more effectively communicated through graphical representations rather than relying predominantly on tables. Some tables appear overly dense and reduce readability; in particular, tables with country rankings could be moved to supplementary materials.

Finally, the conclusions should be more concise and clearly linked to the main findings, with a stronger emphasis on their implications.

Overall, while the study is relevant and potentially valuable, substantial revisions are needed to improve clarity, structure, and scientific presentation.

Reviewer #2: Dear Authors,

Congrats for the manuscript. It has scientific merit, however some clarifications should be made for general audiences who can not be familiar with the technical nature of the topic. For example, added value with this study should be obviously explained in the discussion. You should give clear explanations on why your findings differed from various studies and strengths, and drawbacks with your study design. This revisions will lead your discussion part getting confident with scope. Additionally, there exist plenty of sentences in findings section that can easily go to the discussion, so your own findings should remain in their places. Lastly, during the covid pandemic period, both local studies and comparative studies were published to evaluate excess mortality, so you may consider to discuss your results with their excess mortality data.

Kind regards,

Reviewer

**********

6. PLOS authors have the option to publish the peer review history of their article (what does this mean?). If published, this will include your full peer review and any attached files.

If you choose “no”, your identity will remain anonymous but your review may still be made public.

**Do you want your identity to be public for this peer review?**  For information about this choice, including consent withdrawal, please see our Privacy Policy.

Reviewer #1: **Yes:**  Natalya Glushkova

Reviewer #2: No

**********

[NOTE: If reviewer comments were submitted as an attachment file, they will be attached to this email and accessible via the submission site. Please log into your account, locate the manuscript record, and check for the action link "View Attachments". If this link does not appear, there are no attachment files.]

To ensure your figures meet our technical requirements, please review our figure guidelines: https://journals.plos.org/plosone/s/figures

You may also use PLOS’s free figure tool, NAAS, to help you prepare publication quality figures: https://journals.plos.org/plosone/s/figures#loc-tools-for-figure-preparation.

NAAS will assess whether your figures meet our technical requirements by comparing each figure against our figure specifications.

# Author comment: Author response to Decision Letter 1

**DOI:** 10.1371/journal.pone.0353766.r002

14 May 2026

To the reviewers and editor: Thank you very much for taking the time to read my paper. I will do my best to respond to all the issues raised. Below I provide my responses on a point by point basis.

Reviewer #1: .

1. The abstract in its current form is not sufficiently structured and lacks key quantitative details. While it outlines the general analytical approach, it does not provide specific numerical results (e.g., correlation coefficients, effect sizes, or statistical significance levels), which limits its scientific clarity and impact. A more structured abstract (background, methods, results, conclusions) with clearly reported numerical findings is strongly recommended.

Thank you for this comment. The word limit for the abstract is 300 words, which is why my original abstract was quite concise. In the revised version I have now structured the abstract more formally, using a background/methods/results/conclusions approach. I have also included some numerical results concerning rank correlation coefficients across the different mortality metrics and also with respect to GDP per capita and the Gini coefficient. The revised abstract is still within the 300 word limit.

2. More broadly, the manuscript is difficult to follow due to the lack of adherence to a clear IMRAD structure. The introduction does not adequately establish the relevance and novelty of the study. It lacks a clear articulation of the research gap and does not sufficiently reference key prior studies in the field of excess mortality during COVID-19. Strengthening the justification of the study and situating it within existing literature would significantly improve the manuscript.

Thank you for this comment. The original manuscript adhered loosely to the “Manuscript Organization” outline in the PLOS submission guidelines. I have restructured the manuscript more strictly according to the IMRAD norms.

In response to the second part of this comment, I have reviewed a number of key prior studies and placed my own study in the context of their contribution. I point out that different studies have examined the importance of (a) different estimates of excess mortality (b) different metrics such as years of life lost and (c) the impact of age adjustment. However, to the best of my knowledge there is no paper which addresses all of these issues simultaneously, and my paper fills this gap. In addition, I included other population level factors such as GDP per head and country inequality in my analysis.

3. The methodology section requires clearer and more structured description. In particular, the rationale for selecting the specific dataset and the included countries is not sufficiently explained. It would be important to clarify why these countries were chosen, what makes them comparable or analytically meaningful, and how this selection supports the study objectives and potential generalizability of findings.

Thank you for this comment. In line with the revision to the structure of the paper according to IMRAD norms, I have restructured the methodology section and hopefully it is now clearer. In terms of the choice of measure and countries, there are a number of estimates of excess mortality available and which particular one to choose is very important. Hence I devote some time to this. On balance the K&K choice seemed best. Critically It has estimates of excess mortality by age group, which enables the age adjustment to be made. The choice of countries was dictated by those countries for which the breakdown of excess mortality by age was available and hence this is not a “representative” sample of countries, and I have pointed out that other studies in the field have had to take this approach. I have inserted a number of statements to make it absolutely clear that this sample is thus not in any way “representative”. I make clear in the conclusions section that the extrapolation of results from this sample is also subject to this caveat.

4. The presentation of results could also be improved. Correlation analyses would be more effectively communicated through graphical representations rather than relying predominantly on tables. Some tables appear overly dense and reduce readability; in particular, tables with country rankings could be moved to supplementary materials.

Thank you for this comment. I have removed four tables to supplementary materials S1 Tables: the country rankings for age-specific rankings (originally table 1), the rankings by different mortality metrics (originally table 4) and the two tables listing the clusterings (originally tables 7 and 8). I thought it best not to replace all of these tables with a graph. However, I have inserted six extra graphs into the supplementary material in file S2 Figures which provides the material from the original table 4 in graphical form (I am happy to put these graphs into the main text if requested by referee/editor).

5. Finally, the conclusions should be more concise and clearly linked to the main findings, with a stronger emphasis on their implications.

Thank you. I have rebalanced the Discussion and Conclusion sections. The Conclusion section is now much shorter, and summarises the findings, enters the caveat regarding the sample of countries analysed and stresses that the paper has value in terms of future studies which may examine Covid-related outcomes.

Reviewer #2:

1. It has scientific merit, however some clarifications should be made for general audiences who can not be familiar with the technical nature of the topic. For example, added value with this study should be obviously explained in the discussion. You should give clear explanations on why your findings differed from various studies and strengths, and drawbacks with your study design. This revisions will lead your discussion part getting confident with scope.

Thank you for this comment. This comment overlaps significantly with comment 2 of reviewer 1, hence I refer to my response to that comment. In terms of drawbacks of this study, I have added emphasis on the narrow range of countries included (by data necessity) and stressed that extrapolation of results here to other countries should only be done with great care.

2. Additionally, there exist plenty of sentences in findings section that can easily go to the discussion, so your own findings should remain in their places. Lastly, during the covid pandemic period, both local studies and comparative studies were published to evaluate excess mortality, so you may consider to discuss your results with their excess mortality data.

Thank you for this comment. I have re-balanced the paper, and moved some text from the “Results” section to the “Discussion” section.

In response to the second part of this comment, I have expanded the Introduction section and within that I have included reviews of some of the most relevant comparative studies and compared their approach to mine.

Attachment Submitted filename: Response to Reviewers.docx

# Peer review: Decision Letter 1

**DOI:** 10.1371/journal.pone.0353766.r003

**Authors:** Muhammad Umair, Muhammad Umair

29 Jun 2026

Covid-19 Related Excess Mortality: An Analysis by Age for Selected Countries

PONE-D-26-08812R1

Dear Dr. Madden,

We’re pleased to inform you that your manuscript has been judged scientifically suitable for publication and will be formally accepted for publication once it meets all outstanding technical requirements.

Within one week, you’ll receive an e-mail detailing the required amendments. When these have been addressed, you’ll receive a formal acceptance letter and your manuscript will be scheduled for publication.

An invoice will be generated when your article is formally accepted. Please note, if your institution has a publishing partnership with PLOS and your article meets the relevant criteria, all or part of your publication costs will be covered. Please make sure your user information is up-to-date by logging into Editorial Manager at Editorial Manager® and clicking the ‘Update My Information' link at the top of the page. For questions related to billing, please contact billing support.

If your institution or institutions have a press office, please notify them about your upcoming paper to help maximize its impact. If they’ll be preparing press materials, please inform our press team as soon as possible -- no later than 48 hours after receiving the formal acceptance. Your manuscript will remain under strict press embargo until 2 pm Eastern Time on the date of publication. For more information, please contact onepress@plos.org.

Kind regards,

Muhammad Umair, Ph.D.

Academic Editor

PLOS One

Additional Editor Comments (optional):

Reviewers' comments:

Reviewer's Responses to Questions

**Comments to the Author**

1. If the authors have adequately addressed your comments raised in a previous round of review and you feel that this manuscript is now acceptable for publication, you may indicate that here to bypass the “Comments to the Author” section, enter your conflict of interest statement in the “Confidential to Editor” section, and submit your "Accept" recommendation.

Reviewer #2: All comments have been addressed

Reviewer #3: All comments have been addressed

**********

2. Is the manuscript technically sound, and do the data support the conclusions?

The manuscript must describe a technically sound piece of scientific research with data that supports the conclusions. Experiments must have been conducted rigorously, with appropriate controls, replication, and sample sizes. The conclusions must be drawn appropriately based on the data presented.

Reviewer #2: Yes

Reviewer #3: Yes

**********

3. Has the statistical analysis been performed appropriately and rigorously?

Reviewer #2: Yes

Reviewer #3: Yes

**********

4. Have the authors made all data underlying the findings in their manuscript fully available?

The PLOS Data policy requires authors to make all data underlying the findings described in their manuscript fully available without restriction, with rare exception (please refer to the Data Availability Statement in the manuscript PDF file). The data should be provided as part of the manuscript or its supporting information, or deposited to a public repository. For example, in addition to summary statistics, the data points behind means, medians and variance measures should be available. If there are restrictions on publicly sharing data—e.g. participant privacy or use of data from a third party—those must be specified.

Reviewer #2: Yes

Reviewer #3: Yes

**********

5. Is the manuscript presented in an intelligible fashion and written in standard English?

PLOS ONE does not copyedit accepted manuscripts, so the language in submitted articles must be clear, correct, and unambiguous. Any typographical or grammatical errors should be corrected at revision, so please note any specific errors here.

Reviewer #2: Yes

Reviewer #3: Yes

**********

6. Review Comments to the Author

Please use the space provided to explain your answers to the questions above. You may also include additional comments for the author, including concerns about dual publication, research ethics, or publication ethics. (Please upload your review as an attachment if it exceeds 20,000 characters)

Reviewer #2: Thanks for considering the recommendations from both of the reviewers. The scientific merit has been increased. Good luck for the publication.

Reviewer #3: I have no additional comments for the author, including concerns about dual publication, research ethics, or publication ethics.

**********

7. PLOS authors have the option to publish the peer review history of their article (what does this mean?). If published, this will include your full peer review and any attached files.

If you choose “no”, your identity will remain anonymous but your review may still be made public.

**Do you want your identity to be public for this peer review?**  For information about this choice, including consent withdrawal, please see our Privacy Policy.

Reviewer #2: No

Reviewer #3: No

**********

# Editor report: Acceptance letter

**DOI:** 10.1371/journal.pone.0353766.r004

**Authors:** Muhammad Umair, Muhammad Umair

PONE-D-26-08812R1

PLOS One

Dear Dr. Madden,

I'm pleased to inform you that your manuscript has been deemed suitable for publication in PLOS One. Congratulations! Your manuscript is now being handed over to our production team.

At this stage, our production department will prepare your paper for publication. This includes ensuring the following:

* All references, tables, and figures are properly cited

* All relevant supporting information is included in the manuscript submission,

* There are no issues that prevent the paper from being properly typeset

You will receive further instructions from the production team, including instructions on how to review your proof when it is ready. Please keep in mind that we are working through a large volume of accepted articles, so please give us a few days to review your paper and let you know the next and final steps.

Lastly, if your institution or institutions have a press office, please let them know about your upcoming paper now to help maximize its impact. If they'll be preparing press materials, please inform our press team within the next 48 hours. Your manuscript will remain under strict press embargo until 2 pm Eastern Time on the date of publication. For more information, please contact onepress@plos.org.

You will receive an invoice from PLOS for your publication fee after your manuscript has reached the completed accept phase. If you receive an email requesting payment before acceptance or for any other service, this may be a phishing scheme. Learn how to identify phishing emails and protect your accounts at https://explore.plos.org/phishing.

If we can help with anything else, please email us at customercare@plos.org.

Thank you for submitting your work to PLOS ONE and supporting open access.

Kind regards,

PLOS ONE Editorial Office Staff

on behalf of

Dr. Muhammad Umair

Academic Editor

PLOS One
